# Mammalian body size is determined by interactions between climate, urbanization, and ecological traits

**DOI:** 10.1038/s42003-021-02505-3

**Published:** 2021-08-16

**Authors:** Maggie M. Hantak, Bryan S. McLean, Daijiang Li, Robert P. Guralnick

**Affiliations:** 1 Department of Natural History, Florida Museum of Natural History, University of Florida, Gainesville, FL USA; 2 Department of Biology, University of North Carolina Greensboro, Greensboro, NC USA; 3 Department of Biological Sciences, Louisiana State University, Baton Rouge, LA USA; 4 Center for Computation & Technology, Louisiana State University, Baton Rouge, LA USA

**Keywords:** Climate-change ecology, Evolutionary ecology

## Abstract

Anthropogenically-driven climate warming is a hypothesized driver of animal body size reductions. Less understood are effects of other human-caused disturbances on body size, such as urbanization. We compiled 140,499 body size records of over 100 North American mammals to test how climate and human population density, a proxy for urbanization, and their interactions with species traits, impact body size. We tested three hypotheses of body size variation across urbanization gradients: urban heat island effects, habitat fragmentation, and resource availability. Our results demonstrate that both urbanization and temperature influence mammalian body size variation, most often leading to larger individuals, thus supporting the resource availability hypothesis. In addition, life history and other ecological factors play a critical role in mediating the effects of climate and urbanization on body size. Larger mammals and species that utilize thermal buffering are more sensitive to warmer temperatures, while flexibility in activity time appears to be advantageous in urbanized areas. This work highlights the value of using digitized, natural history data to track how human disturbance drives morphological variation.

## Abstract (web summary)

Anthropogenically-driven climate change is responsible for body size decreases in mammals. Using an important dataset of historically-collected data and data from continental-scale survey efforts from the National Ecological Observatory Network, Hantak et al. show that urbanization plays an important role in mediating this dynamic.

## Introduction

Body size is an easily measured, integrator trait that scales with many ecological characteristics of organisms^1–3^. Because of this, understanding drivers of body size variation has been a central goal of ecology over the last half century^4–6^. Macroscale studies of body size across broad environmental gradients date back to the seminal work of Carl Bergmann^7^ (i.e., Bergmann’s Rule—the tendency for larger organisms to be found in cooler climates), although with much subsequent debate about the generality of patterns and underlying mechanisms^8–10^. Some species—but not all—follow responses to temperature predicted by Bergmann, with smaller average body sizes in warmer climates. Food availability can also strongly determine species’ body size differences^11,12^.

Much less attention has been paid to anthropogenic influences on body size that play out at the local or regional scale (but see^13^), which provides a distinct set of challenges and opportunities for organisms. For example, while urbanization may increase potential for novel human-caused conflict and predation, these novel environments can also lead to decreased predation rate^14^ and increased food resources. The complexity of urban environments provides an opportunity to examine species responses to a variety of major ecological gradients in real time, and to test the applicability of longstanding ecogeographic rules within the human-built environment. For example, Ives et al.^15^ found Australian cities harbor a large number of threatened plants and animals, which may be due to a high amount of landscape heterogeneity (e.g., plant cultivation) in urban areas.

Body size variation due to human alteration of landscapes may be driven by multiple possible, non-mutually exclusive drivers. First, due to human activity and built infrastructure, cities are generally warmer than surrounding areas, a phenomenon known as the urban heat island effect^16^. Animals inhabiting warmer urban heat islands are predicted to be smaller in body size based on the general tendency for species to decrease in size with increasing temperature^17–20^. There is empirical support for urban heat island effects driving decreases in body size in various animal taxa, namely insects^21,22^, but limited support in endotherms^23^. Second, heterogeneity in urban areas can contribute to increased food resources and water availability compared to rural areas^24^, which could further mediate body sizes in urban areas (i.e., a resource rule^25^). Finally, Schmidt and Jensen^26,27^ suggested that species that experience landscape fragmentation driven by urbanization and an increased human footprint should either go extinct or adapt through changes in traits, namely increasing body size for smaller species and decreases for larger species. Each of these hypotheses have clear, alternate predictions about the overall effects of urbanization, and can be emplaced in the broader context of overall climatic gradients.

Mammals represent a good study system for examining the potentially multifaceted effects of climate and urbanization on body size because they are ecologically diverse, exhibit a wide range of body sizes, have diverse life history strategies, and are well-represented in biodiversity datasets. Mammals have evolved to fill a large variety of niches including aquatic, terrestrial, and even subterranean habitats, often facilitated by the evolution of key functional, morphological, or behavioral traits^28^. We expect that these traits strongly mediate current and future responses of organisms to climate variation inside and outside of urban areas; however, few studies have directly examined how these factors may influence spatiotemporal trends in recent global responses of mammals (but see^29–31^). Hibernation, a suite of behaviors such as nocturnality, or spending portions of the life cycle underground (i.e., habitat buffering), may be critical for coping with unsuitable climatic conditions especially in the short term^32,33^. Finally, mammals are well-sampled in many biodiversity datasets, with body size measurements often taken in the field as part of long-standing collection practices. This creates an opportunity to analyze body size across an entire vertebrate clade, and to establish robust workflows for dealing with spatiotemporal collecting biases which need to be carefully considered in downstream modeling.

**Fig. 1:**
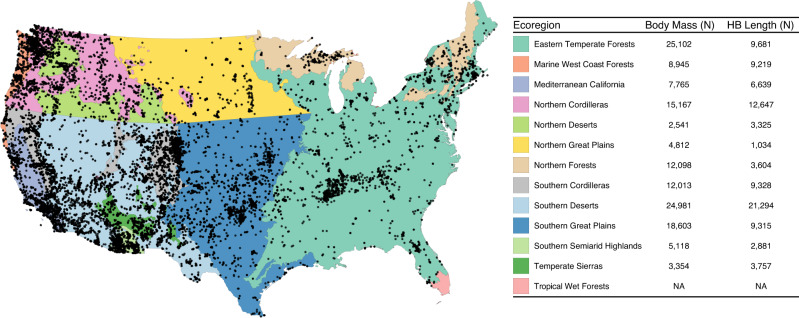
Body mass and head-body (HB) length record localities. Designated spatial ecoregions are colored and the key shows the total number of body mass and head-body (HB) length records from each ecoregion. This map was created with R version 3.6.2 (https://www.r-project.org/).

In this study, we compiled multiple datasets containing 140,499 mass and body length records spanning more than 100 mammal species to address broad-scale spatial trends of mammalian body size (Fig. 1, S1, Supplemental Data 1). Our overarching question is how climate and human population density, a proxy for the human built environment (i.e., urbanization; e.g.,^34^), impact mammal body size. We first addressed the relationship between body mass and head-body length, as each is commonly used as a body size metric but the former can vary seasonally due to age, reproductive status, or food availability^35^, potentially weakening mass-length allometries at range-wide scales^36^. We then use a hierarchical modeling framework to identify the main drivers of body size variation, accounting not only for climate and urbanization but also broad differences in habitat and species-specific trends. Drawing on Bergmann’s Rule, we predicted that temperature would negatively impact both metrics of body size (i.e., smaller size in warmer temperatures). We also expected that increasing human population density would drive smaller body size due to heat island effects, thereby amplifying Bergmann’s-like patterns. Alternatively, and given recent results from single species studies (e.g.,^36^), it may also be that body mass increases while body length decreases in urban areas as increased anthropogenic food availability (e.g., garbage or human provisioning of food) allows for more weight^6,37,38^ but need for crypsis or heat island effects drive decreasing length. Further, urbanized areas may mimic islands given their fragmented habitats, driving larger species to decrease in size and smaller species to increase in size.

To develop a more integrative framework for understanding body size variation in mammals, we extended our work to incorporate ecological traits that are related to species thermal biology, which are likely to modify and interact with both of these drivers, especially ability to buffer thermal environments. Therefore, we predicted weaker body size responses from species that hibernate or use torpor and species that utilize habitat buffering (i.e., use of underground or cave habitats) as they are able to avoid extreme climates (e.g.,^39^; but see^40^). We also considered traits such as activity time and expected that nocturnal mammals should increase in size more than diurnal species in urban areas since they can more easily avoid humans but still benefit from food resources. Lastly, based on the hypothesis of more fragmented, island-like habitats in human built environments, we predicted that larger species may decrease in body size and smaller species increase in size in areas of higher human population density. Small size is also predicted to be favored as a greater number of microhabitats are available to escape unfavorable temperatures and avoid human detection^41–43^.

## Results

Aggregation of data across multiple sources (VertNet, NEON, NACSM) generated a significant dataset to examine spatially-structured changes in mammal body mass and head-body length in relation to climate, human population density, and key ecological traits. The best model of body mass variation included the following covariates: Mean Annual Temperature (MAT), Mean Annual Precipitation (MAP), season, sex, human population density, all traits, and all interactions besides population density × small/large mammals (partial *R*^2^ = 0.97). Significant main effects include MAT, MAP, season, sex, human population density, traits of hibernation, activity time, and small/large mean binned body mass (Table 1). This model also included strong interactive effects between MAT, population density, and traits.

**Table 1:** Fixed effect coefficients for the top body mass and head-body length phylogenetic generalized linear mixed models (PGLMMs).

Term	Body mass	Head-body length
(Intercept)	**2.477 (2.107, 2.848)**	**2.317 (2.181, 2.452)**
MAT	**−0.061 (−0.069, −0.054)**	**−0.018 (−0.021, −0.015)**
MAP	**−0.001 (−0.002, −0.001)**	**−0.001 (−0.001, 0.000)**
season:spring	**0.025 (0.024, 0.027)**	**0.008 (0.007, 0.009)**
Season:summer	**0.013 (0.012, 0.014)**	**0.002 (0.002, 0.003)**
Season:winter	**−0.007 (−0.009, −0.005)**	0.000 (−0.001, 0.001)
Sex:male	**0.002 (0.002, 0.003)**	
Population density	**0.008 (0.006, 0.009)**	**0.007 (0.005, 0.008)**
Hibernation:hibernator	0.302 (−0.002, 0.605)	0.033 (−0.072, 0.139)
Hibernation:none	**0.539 (0.169, 0.908)**	**0.164 (0.035, 0.293)**
Habitat buffering:none	0.013 (−0.220, 0.246)	0.015 (−0.067, 0.097)
Habitat buffering:obligate	0.116 (−0.182, 0.415)	0.033 (−0.072, 0.138)
Activity time:diurnal	0.136 (−0.119, 0.392)	0.037 (−0.053, 0.128)
Activity time:nocturnal	**−0.185 (−0.327, −0.044)**	**−0.067 (−0.118, −0.015)**
Small/large body size:small	**−1.157 (−1.383, −0.931)**	**−0.345 (−0.439, −0.251)**
MAT × small/large body size:small	**0.024 (0.019, 0.030)**	**0.005 (0.003, 0.007)**
Population density×small/large body size:small		**−0.003 (−0.004, −0.002)**
MAT × population density	**−0.003 (−0.003, −0.002)**	
MAT × hibernation:hibernator	**0.008 (0.004, 0.011)**	**0.007 (0.005, 0.008)**
MAT × hibernation:none	**0.019 (0.017, 0.021)**	**0.009 (0.008, 0.010)**
MAT × habitat buffering:none	**0.026 (0.021, 0.030)**	**0.007 (0.005, 0.009)**
MAT × habitat buffering:obligate	**0.016 (0.012, 0.021)**	**0.003 (0.001, 0.005)**
Population density × activity time:diurnal	**−0.014 (−0.017, −0.012)**	**−0.006 (−0.007, −0.004)**
Population density × diurnal/nocturnal:nocturnal	**−0.002 (−0.003, −0.001)**	**−0.007 (−0.007, −0.006)**

Bold indicates significant effects; coefficients where the 95% Bayesian credible interval (in parentheses) does not overlap zero.

**Fig. 2:**
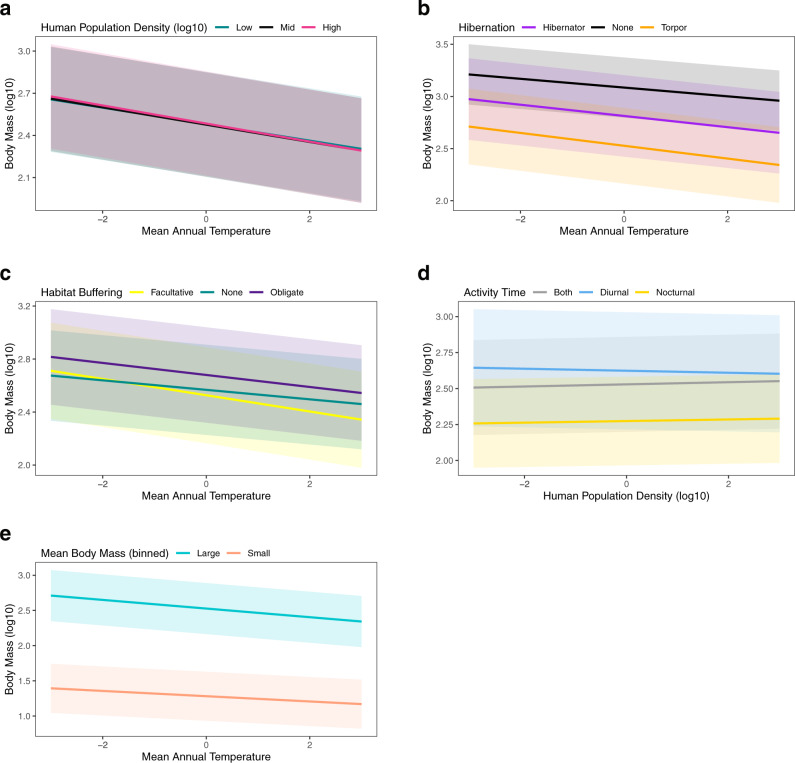
Mammal body mass interactions. Mammalian body mass is influenced by the interaction between (**a**) human population density and mean annual temperature; (**b**) hibernation ability and mean annual temperature; (**c**) habitat buffering and mean annual temperature; (**d**) activity time and human population density; (**e**) and large/small size and mean annual temperature. Predictor variables (mean annual temperature and human population density) were mean-centered and standardized. Error bars in each plot represent 95% credible intervals.

The negative interaction between MAT and population density implies that while mammal body mass increases with decreasing MAT in general, this trend is stronger in areas with higher densities of humans (Estimate = −0.003, 95% Bayesian credible intervals (CI) = −0.003 – −0.002; Table 1, Fig. 2a). Ecological traits also strongly mediated responses of body mass to climate and urbanization. With increasing MAT, species with any hibernation ability and non-hibernators decrease in body mass, but the strength of the decrease is stronger for species that use torpor compared to species that hibernate (Estimate = 0.008, 95% CI = 0.004 – 0.011) and that do not hibernate (Estimate = 0.019, 95% CI = 0.017 – 0.021; Table 1, Fig. 2b). Species that use habitat buffering (obligate and facultative) and non-buffered species decrease in body mass with increasing MAT, but the strength of the decrease is stronger for species that facultatively use habitat buffers compared to species that do not use habitat buffers (Estimate = 0.026, 95% CI = 0.021 – 0.030) and species that are obligated to use buffers (Estimate = 0.016, 95% CI = 0.012 – 0.021; Table 1, Fig. 2c). Diurnal species are larger in body mass and decrease in mass with increasing population density (Estimate = −0.014, 95% CI = −0.017 to −0.012; Table 1, Fig. 2d), compared to nocturnal species or those scored as “both” (Table 1, Fig. 2D). Both large and small mammals (binned mean size) decrease in body mass with increasing MAT, but the strength of the decrease is stronger for larger species (Estimate = 0.024, CI = 0.019 – 0.030; Table 1, Fig. 2e).

When examining head-body length as a body size metric, the best-fit model consisted of MAT, MAP, season, population density, all traits, and all interactions except MAT×human population density (partial *R*^2^ = 0.97). Significant single predictors are MAT, MAP, season, human population density, hibernation, activity time, and small/large mean binned head-body length (Table 1).

**Fig. 3:**
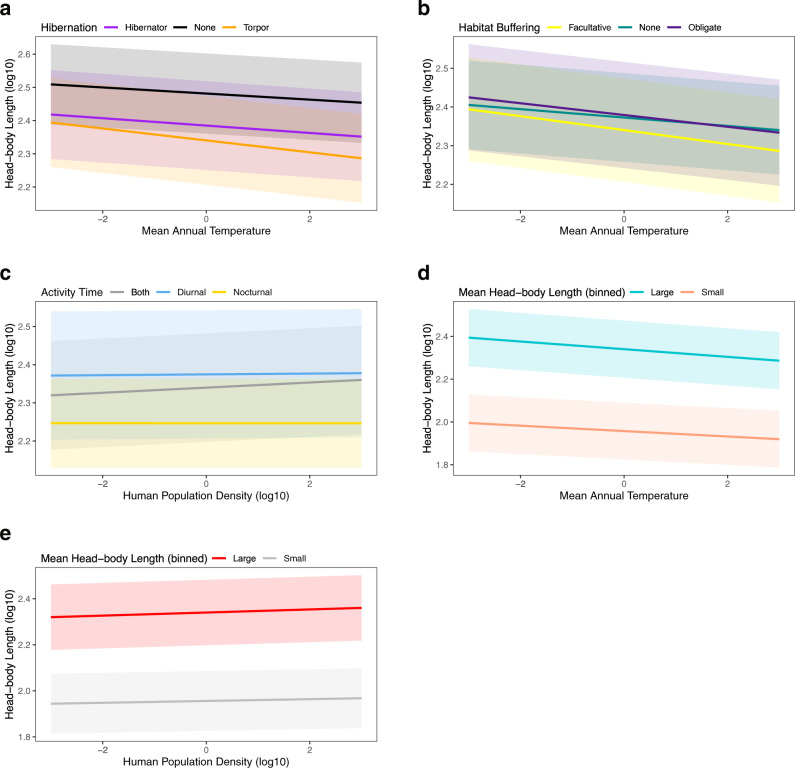
Mammal head-body length interactions. Mammalian head-body (HB) length is influenced by the interaction between (**a**) hibernation ability and mean annual temperature; (**b**) habitat buffering and mean annual temperature; (**c**) activity time and human population density; (**d**) large/small size and mean annual temperature, and (**e**) large/small size and human population density. Predictor variables (mean annual temperature and human population density) were mean-centered and standardized. Error bars in each plot represent 95% credible intervals.

Similar to body mass, we find strong interactive effects between MAT and population density with traits. Head-body length is negatively correlated with MAT for species with any hibernation ability and non-hibernators, but the strength of the decrease is stronger for species that use torpor compared to species that hibernate (Estimate = 0.007, 95% CI = 0.005–0.008) and that do not hibernate (Estimate = 0.009, 95% CI = 0.008–0.010; Table 1, Fig. 3a). Species that utilize habitat buffering (obligate and facultative) and non-buffered species decrease in body mass with increasing MAT, but the strength of the decrease is stronger for species that facultatively use habitat buffers compared to species that do not use habitat buffers (Estimate = 0.007, 95% CI = 0.005–0.009) and species that are obligated to use habitat buffers (Estimate = 0.003, 95% CI = 0.001–0.005; Table 1, Fig. 3b). Nocturnal species decrease slightly in head-body length with increasing population density (Estimate = −0.007, 95% CI = −0.007 to −0.006), whereas diurnal species and species that display both tendencies increase in head-body length with increasing population density, but the strength of the increase is weaker for diurnal species (Estimate = −0.006, 95% CI = −0.007 to −0.004; Table 1, Fig. 3c). The effect of the decrease in head-body length with increasing MAT is stronger for larger mammals compared to smaller species (Estimate = 0.005, 95% CI = 0.003–0.007; Table 1, Fig. 3d). Large and small mammals increase in head-body length with increasing population density, but the strength of the increase is stronger for larger mammals (Estimate = −0.003, 95% CI = −0.004 to −0.002; Table 1, Fig. 3e).

## Discussion

Climate as a driver of animal body size variation has been well documented across both space and time^18,44,45^. However, a myriad of anthropogenic global effects (e.g., habitat degradation and fragmentation, pollution) are known to impact organisms at both local and regional scales, promoting complex responses that may be difficult to contextualize with regard to longstanding ecogeographic rules. Further, these responses likely vary among species and clades because ecological traits mediate exposure and thus the intensity of changes experienced. Here, we investigate how climate (a more constant driver over earth history) and urbanization (a novel disturbance) influence mammalian body size, and how life history and other ecological traits mediate those effects. We test these ideas by utilizing hundreds of thousands of compiled mammal body size records from natural history collections and field censuses, spanning 80 years and over 100 North American species.

Despite nearly two centuries of work examining the links between climate and body size, we found that both human population density (a proxy for urbanization) and temperature are important predictors of mammalian body size variation. Finding weak support for interspecific Bergmann’s Rule, Gohli and Voje^46^ suggested that other variables, besides temperature and latitude, are more important drivers of mammalian body mass. However, few studies have tested broad-scale effects of urbanization on body size across mammal species. Here, in all cases, the main effect of increased urbanization was larger body size, consistent with mammals benefiting from increased food resources, higher calorie diets, ecological release (i.e., from predators and competitors), or all three^13,47–49^ (but see^50^). We found no clear evidence for urban heat island effects on body size. We had considered that interactions between climate and urbanization could mean that heat island effects might only be present in the coldest areas. But here as well, we found the opposite—mammals in urbanized, cold areas have larger, not smaller, body masses than their rural counterparts, a result that likely speaks to more available food in urban areas. While a few studies have found support for urban heat island effects leading to reductions in body size in ectotherms^21,51^, there is currently no evidence of mammals following this trend. The overall result across all mammals examined is that head-body lengths are greater in urban areas regardless of temperature. Our study does not account for intra-urban variation in land use that can influence heat island pockets (e.g.,^52^); as such, finer-scale investigations of the relationship between surface characteristics with temperature and body size may more precisely demonstrate the role of heat islands in impacting body size.

Our results suggest that one key outcome of urbanization is provisioning of novel, reliable food resources. Yom-Tov^53^ found a similar result for carnivoran body size; increased body size was related to increased anthropogenic food sources and not temperature. In addition to increased food, cities provide reliable water resources and shelter by use of built structures, which might decrease energetic costs and enhance growth rate and body condition^54^. Based on our results, the one exception to this pattern is that body mass was nearly equivalent among levels of urbanization in the warmest areas. It is possible that a temperature threshold exists above which increased body size becomes less energetically advantageous (regardless of available food). This pattern may also emerge if constant food availability permits survival in milder winters where fat reserves are less critical, potentially also aiding quicker locomotor movements to escape predation or reductions in foraging time^55–58^. Future studies quantifying food availability between spatially distinct regions are warranted as some mammals appear to be adapting to novel food resources in urbanized areas^59^. In addition, as agricultural or other non-urbanized areas can also reliably provide substantial food resources to mammals^6^, future work should examine variation in body size across different land use classes.

We acknowledge that several mammal species may be urbanophobic or unable to exploit resources provided in urban areas. Our strict filtering criteria limited analyses to abundant and well-collected mammal species, but these species are likely to be urbanophilic or urban-neutral given that many collections are near human-populated areas. Thus, our combined results do not necessarily apply to all North American mammal species, and it is known that the percentage of urbanized area plays a role in determining which species occupy those areas^60^. Ultimately, life history strategies, as well as morphological traits, facilitate the ability to occupy urban environments, and filter out species lacking suitable characteristics^61–63^. Thus, species inhabiting the most urbanized areas are likely those with suites of traits that allow utilization of the novel resources in cities. Even so, Parsons et al.^64^ found no difference in species diversity or richness along an urban-wildland gradient, and suggested mammals likely adapted to developed areas over the last few decades. Further studies investigating species occupancy across developed gradients will help elucidate adaptive trait responses to human-dominated landscapes.

Species traits directly related to thermoregulation and energetics appear to play an integral role in mediating the effects of climate and urbanization on body size, but not in the directions we predicted from theory. We predicted species that utilize thermal buffering (habitat buffering or hibernation ability) would show weaker responses of body size change in warmer regions as these traits allow for avoidance of unfavorable climatic conditions^65^. In contrast, we found species that use these behaviors are more sensitive to warmer temperatures than non-thermally buffered species, and respond to warmer temperatures with stronger decreases in body size. Thus, for species that hibernate or undergo torpor, exposure to temperatures during the active periods alone may still be a sufficiently strong selective pressure. Further, species that experience torpor were the most sensitive to variation in temperature. This sensitivity may be due to differences in the circadian clock or metabolic rate in species that use torpor compared to hibernators^66^. For species that use habitat buffering (facultatively or obligates), lack of sufficient microhabitat heterogeneity due to extreme climates, clearcutting of forests, or increases in forest fires can result in decreased variation in ambient temperatures between exposed and buffered areas and ultimately reduce the effectiveness of that behavior^42,67^. Further work to better understand physiological tolerances of species that use thermal buffering in relation to patterns of global environmental gradients are necessary, as these relationships are complex and likely involve multiway interactions between landscape change, climate change, and ecological traits^40,68^.

Daily activity pattern represents another important trait for adaptation to variable environments. Flexibility in activity times appears to be advantageous in more urbanized areas. McCain and King^30^ found mammals that can switch between diurnality and nocturnality were least likely to respond negatively or respond at all to climate change, and postulated this was due to the ability of these species to select climatic conditions that are suitable for activities. Relative to mammals that are flexible in their activity times, we found diurnal species decrease in body mass, but increase in head-body length with increasing urbanization. An elongated body form may represent a locomotory adaptation, allowing diurnal mammals to exploit more shelters (e.g., burrows^69^). With increasing urbanization, nocturnal mammals demonstrate a minimal decrease in head-body length, but increase in mass similar to species that are active anytime. Decreases in head-body length could be suggestive of an adaptive response to avoid detection (i.e., crypsis^36^), while increasing mass is indicative of nocturnal mammals benefiting from increased food resources in urban areas. The same idea may hold for species that are able to selectively avoid human detection by being flexible in activity times.

Finally, our results provide new insight into average body size itself as a trait that can modulate responses to changing environments. In areas with warmer temperatures, we found larger mammals decrease in size more than smaller mammals. This result is in contrast to the meta-analysis of Ashton et al.^8^, who found no difference between small or large mammals. However, a reanalysis of that dataset demonstrated no general tendency for small mammals to increase or decrease in size, while larger mammals tended to display a Bergmann’s-like response^70^, consistent with our results. In another meta-analysis of 73 North American mammal species, McCain and King^30^ found the largest mammals examined were 27 times more likely to respond to climate change compared to the smallest mammals. These previous studies are all limited in that they are meta-analyses (also see^71^), vary in statistical approach, and do not leverage the dense intraspecific sampling we achieved here. Our work draws strength from the use of a single hierarchical modeling framework for separate measures of body mass and head-body length and reveals a robust signal of larger mammals being more sensitive to variation in temperature, and conforming to Bergmann’s Rule. Lastly, small or large size does not mediate variation in body mass with increasing urbanization. However, we did find large and small mammals increase in head-body length, but large mammals increase to a greater extent in more urbanized areas. These results do not lend support to the Island Rule, from which we would expect body size homogenization with increasing urbanization^26^. Instead, increasing length (especially for larger species) may aid movement across fragmented landscapes^72^.

In this work, we have focused primarily on the utility of digital biodiversity datasets such as natural history collections and ecological monitoring efforts to examine spatial trends in mammal body size. However, we recognize that temporal changes may also be inherent given well-known climate and urbanization changes over the timescale of our dataset. We explicitly fit a decadal random term to control for this variation, but the constituent datasets themselves are also temporally structured, complicating issues with controlling for methodological issues^36^. One future possibility is to add a spatially controlled time-series, which would provide a strong basis for examining temporal trends across multiple sites. In addition, finer-scale regional or community-level ecological studies would provide a more detailed understanding of the drivers of temporal changes^73^.

Our understanding of how human-mediated pressures impact mammalian body size has remained limited for decades, and is often tied to simplistic ecogeographic “rules”, whose validity continues to be called into question^10^. Our data-intensive work showcases the importance of incorporating other human disturbances beyond climate variation, and also reflects how multiple pressures interact with species traits to influence differences in body size. Beyond the finding that urbanization has a strong impact on body size, it is surprising that species with thermal buffering traits are more sensitive to temperature. This has major implications for conservation management of native species and suggests that these species are under increasingly intense selection not just for parameters such as phenology, but also morphological traits like body size. Further collection and digitization of trait data at the individual level remains essential for improved understanding of macro-scale spatiotemporal patterns of body size variation, especially given accelerating climate warming and urbanization^74–76^.

## Methods

### Data sources & aggregation

We obtained mammal body size data from three repositories: VertNet^77^, the National Ecological Observatory Network (NEON^78^; https://www.neonscience.org/), and the North American Census of Small Mammals (NACSM^79–87^). Standard body mass and total body length measures were extracted from the VertNet corpus following the approach of Guralnick et al.^88^. NEON data were obtained using the *neonUtilities* R package^89^, but only body mass was used from NEON survey events because accurate length measures are difficult to obtain on live, unanesthetized, mammals^36^. We examined body mass variation across sources and found no systematic biases of measures from NEON or other sources. NACSM data were obtained via manual digitization from published reports, and were extracted for a subset of species that had body size measurements and which were also obtained from VertNet and NEON. We aggregated VertNet data with corresponding species from NEON and NACSM and harmonized data field names across the three sources. Any migratory species were removed as they can experience a wide breadth of environmental conditions. Measures of head-body length were then derived by subtracting tail length from total length for each individual. As a preliminary step, we filtered the data to those species with a minimum of 100 records for body mass or length.

### Data filtering

Additional filtering included removal of records lacking; (1) latitude and longitude; (2) sex, including those with ambiguous sex assignments (e.g., “female?”); (3) date information—we required month, day, and year for each record. However, for some specimen records with missing locality, we first aimed to manually georeference data when possible using the protocols of Chapman and Wieczorek^90^, which uses a combination of Google Maps (https://www.google.com/maps) and the MaNIS georeferencing calculator^91^ (http://manisnet.org/gci2.html). Manual curation based on locality was also necessary in some instances. For example, several records of *Canis lupus* came from zoos or sanctuaries; all zoo records were removed by hand. We next created two additional fields from the record dates, “season collected” and “decade”. Month of collection was used to bin the records into spring (March-May), summer (June-August), fall (September-November), and winter (December-February) seasons. In some species, tail length is not reported due to very small or missing tails, and in those cases we relied on total length. We also filtered juveniles from the dataset based on age assignments in the Darwin Core field “lifeStage” (for VertNet) or based on body size measurements below a lower threshold for each individual species based on literature searches and reputable online databases (see Supplemental Data 1). To remove any additional erroneous data values (e.g., digitization errors), we used a 95% dispersion-based threshold using the *OutlierDetection* R^92^ package^93^. Taxonomy was updated for all records to ensure scientific names were synonymous across data sources.

### Relationship between body mass and head-body length

We ran simple univariate linear regressions where log_10_ head-body length predicts log_10_ body mass for each species. Correlations were generally weak among species as indicated by the vast majority of the fits with *r*^2^ < 0.5 (Supplemental Data 1). As such, we compiled two body size datasets: body mass and head-body length which were used as response variables in separate downstream models.

### Population density and climate

As a proxy for urbanization intensity, we used high-resolution (1 × 1 km) decadal human population density data for the conterminous USA (years 1940–2010) from Fang and Jawitz^94^. We selected human population density over impervious land cover or Human Footprint Index^95^ as our measure of urbanization because it more directly accounts for anthropogenic effects (e.g., food waste) and encompasses the range of mammalian species collection dates used in this study. Our work follows others, e.g., Li et al.^96,97^, who make similar arguments as to the value of human population density as a proxy for urbanization. We annotated each record with human population density data by first aggregating density data to a resolution of 10 ×10 km and indexing this value by decade collected and record locality. We chose coarser human population density spatial resolution given uncertainties in georeferencing and in order to provide a reasonably broad human population context. Historical climate data were obtained from the PRISM Climate Group^98^ at 4 km resolution for both historical and contemporary body size observations. We extracted mean annual temperature (MAT) and mean annual precipitation (MAP) from PRISM based on observation year and geocoordinates.

### Spatial regions

To control for habitat differences across our region of interest, and to account for sample distribution (Fig. 1, S1), we included ecoregional membership as a random effect in each model. We used the United States Environmental Protection Agency (EPA) Level 1 ecoregions (https://www.epa.gov/eco-research/ecoregions), but further divided three ecoregions given the large climate and latitudinal range. We split the ‘Great Plains’, ‘Northwestern Forested Mountains’, and ‘North American Deserts’ ecoregions at 42 degrees latitude and renamed the ecoregions: ‘Northern and Southern Great Plains’, ‘Northern and Southern Cordilleras’, and ‘Northern and Southern Desserts’, respectively (Fig. 1).

### Phylogeny and mammal traits

We obtained a global mammal consensus phylogeny from Upham et al.^99^ (http://vertlife.org/data/mammals/) and pruned the tree to match the species present in the two datasets (body mass, *n* = 101; head-body length, *n* = 99). We also compiled ecological traits for the final species sets that likely influence body size response to environmental variation (Fig. S2, Supplemental Data 1). These traits include hibernation ability (hibernation, torpor, or neither), habitat buffering ability (obligate, facultative, or neither), daily activity pattern (diurnal, nocturnal, or both), and average body size binned into small (<500 g; <200 mm) and large (>500 g; >200 mm) categories (Fig. S2, sources provided in Supplemental Data 1). Sensitivity analyses of mean average body size binned differently (e.g., ranging 450–550 g, 150–250 mm) yielded the same model results. Additional detail and supporting references for species trait classifications can be found in the supplementary materials (Supplemental Methods, Fig. S2, Supplemental Data 1).

### Statistics and reproducibility

To examine drivers of mammalian body size variation, we initially used linear mixed-effects models (LMM), using the R package *lme4*^100^. We log_10_ transformed measures of body mass and head-body length as mammal body size ranges vary by orders of magnitude^101^. In addition, we log_10_ transformed human population density and log transformed MAP to normalize data. We mean-centered and standardized all continuous predictors to have standard deviations of 1, except decade, which we treated as a numeric variable that starts at zero. No continuous model predictors were highly correlated (Fig. S3, S4). All models were run separately for body mass and head-body length.

We ran a set of global models that included fixed effects of MAT, MAP, sex, season collected (spring, summer, fall, and winter), human population density, and the traits listed above (‘Mammal traits’). Inclusion of traits as fixed effects allowed us to directly model their impact on size across the mammalian body size spectrum^102^, as well as how these traits interact with climate and urbanization. Specifically, we examined the interactions of MAT × human population density, MAT × hibernation ability, MAT × habitat buffering, MAT × small/large mammals, population density × activity time, and population density × small/large mammals. We included three random intercepts of ecoregion, decade, and species. The decade random effect was added to our models to account for temporal autocorrelation. We also ran models without the decade random effects, and models with temperature and precipitation averaged over two years and five years. Results were consistent across models with these various combinations of annual temperature averages (data not shown).

After running each global model, we used backward stepwise selection with the step function in the R package *lmerTest*^103^ to find the best-fit model. We checked residuals of the final models, with binary ecological traits of hibernation and habitat buffering and minus the random effect of decade (due to matrix complexity), and found no evidence of spatial autocorrelation (Fig. S5). In addition, we checked model variance inflation factors of predictors to ensure there was no multicollinearity. Marginal and conditional R^2^s were obtained for the best-fit models using the R package *MuMIn*^104^.

To account for potential effects of evolutionary history in these models, we re-ran the best-fit body mass and head-body length models using phylogenetic generalized linear mixed models (PGLMMs) using the R package *phyr*^105^. We used the pruned global mammal consensus phylogeny^99^, described above, to fit the body mass and head-body length PGLMMs using a Bayesian framework. PGLMM and LMM results were largely concordant (Table 1, S1), but differed slightly. We present the PGLMM results in the main text and LMM model results in the Supplemental Material (Table S1). We measured the goodness of fit for the top body mass and head-body length PGLMMs using the R package *rr2*^106^.

### Reporting summary

Further information on research design is available in the Nature Research Reporting Summary linked to this article.

## Supplementary information

Transparent Peer Review File

Supplementary Information

Description of Supplementary Files

Supplementary Data 1

Reporting Summary

## Data Availability

Data used in this study are available at Github (https://github.com/mhantak/Mammal_spatial).

## Code availability

Code used in this study are available at Github (https://github.com/mhantak/Mammal_spatial).
