# Supplementary material for: Mammalian body size is determined by interactions between climate, urbanization, and ecological traits
Source: Commun Biol. 2021 Aug 16;4:972. doi: 10.1038/s42003-021-02505-3 (PMC8367959; doi:10.1038/s42003-021-02505-3)
Supplement: Supplementary file 2 — Supplementary Information [file 42003_2021_2505_MOESM2_ESM.pdf]

Mammalian body size is determined by interactions between climate, urbanization, and ecological traits

Maggie M. Hantak[1,*], Bryan S. McLean[2], Daijiang Li[3,4], Robert P. Guralnick[1,*]

[1]Department of Natural History, Florida Museum of Natural History, University of Florida, Gainesville, FL, USA
[2]Department of Biology, University of North Carolina Greensboro, Greensboro, NC, USA
[3]Department of Biological Sciences, Louisiana State University, Baton Rouge, LA, USA
[4]Center for Computation & Technology, Louisiana State University, Baton Rouge, LA, USA

[*]Corresponding authors: Maggie M. Hantak, maggiehantak@gmail.com, +1 216-548-0951; Robert Guralnick, robgur@gmail.com, +1 352-273-1980

SUPPLEMENTAL METHODS

Ecological Trait Classifications

Hibernation: We coded species physiological capabilities for thermal buffering; specifically, the use of seasonal torpor or hibernation. Species were considered capable of torpor if bouts of inactivity were short, ranging up to approximately two days. Species were considered capable of hibernation if bouts of inactivity were longer, ranging from several days to months. Physiological assignments were made using independent literature searches, including previously published reviews (Ruf and Geiser 2015).

Habitat buffering: We coded species' capabilities for thermal buffering via habitat partitioning; specifically, obligatory (or nearly obligatory) use of underground or cave habitats to modulate ambient temperature. Species were considered subterranean if they spend all (or the vast majority) of their lives underground, including during foraging and reproduction. Species were considered fossorial if they make use of burrows, underground nests, or cave systems at specified periods of each day or year. We coded whether use of thermal buffering options was obligate or not (e.g., a species that nests in a burrow **or** in a more exposed area, a species that can roost in a cave **or** in a more exposed area). Species with facultative buffering strategies use underground retreats but are not reliant upon it. Species were considered terrestrial if they do none of these, and are thus largely exposed to ambient temperatures. Aquatic or semi-aquatic species were scored only based on use of habitat use strategies, not the amount of time spent in water *per se*.

Species ecological trait classifications are shown in Figure S2 and Table S1.

Ruf, T. and Geiser, F., 2015. Daily torpor and hibernation in birds and mammals. *Biological Reviews*, *90*(3), pp.891-926.

SUPPLEMENTAL FIGURES

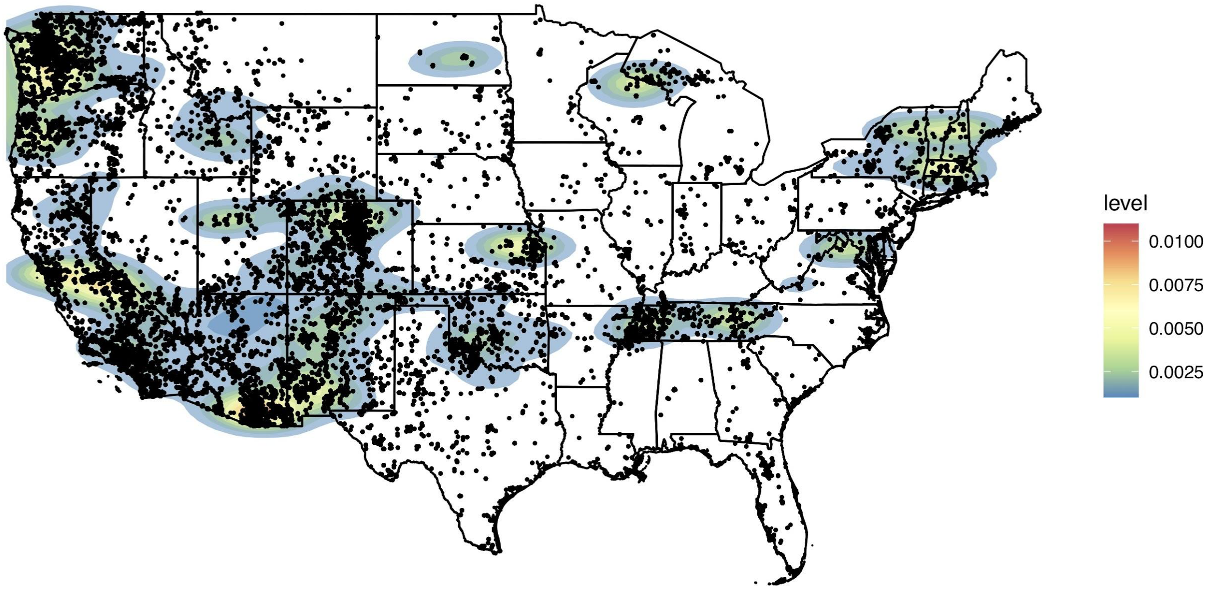

Fig. S1. Heat map of mammal record densities.

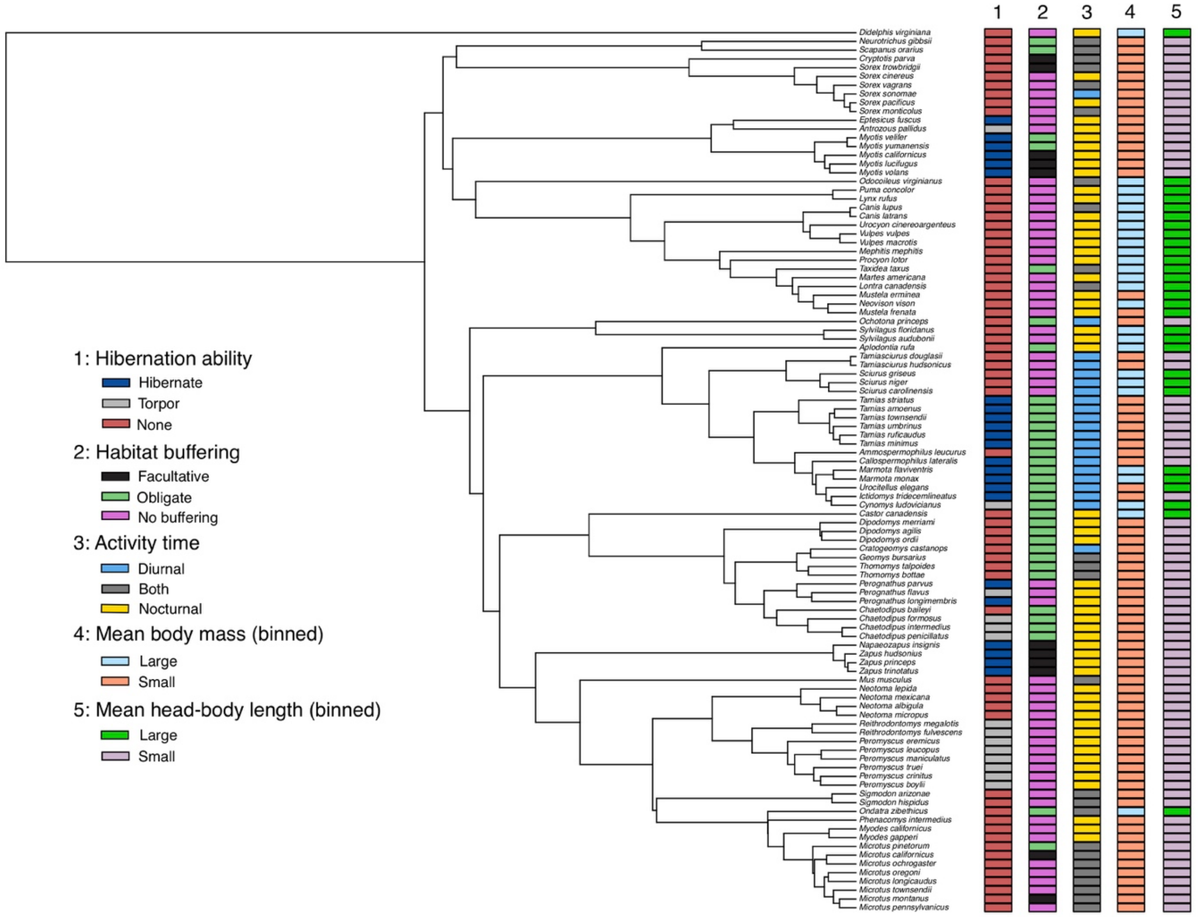

Fig. S2. Phylogeny of study species trimmed from the tree of Upham et al. (2019; http://vertlife.org/data/mammals/) including traits used in this study. Hibernating species include those that experience daily torpor or more prolonged hibernation bouts. Mammals that utilize habitat buffering include species that are fossorial, volant, and subterranean, while non-buffered species in our study are terrestrial and arboreal. Mammal species can be active at night, during the day, or during all times. Species with a large mean body size are >500g; >200mm, while small species are <500g; <200mm. Subset phylogenies of species in separate body mass and head-body length data datasets were used in the PGLMM analyses.

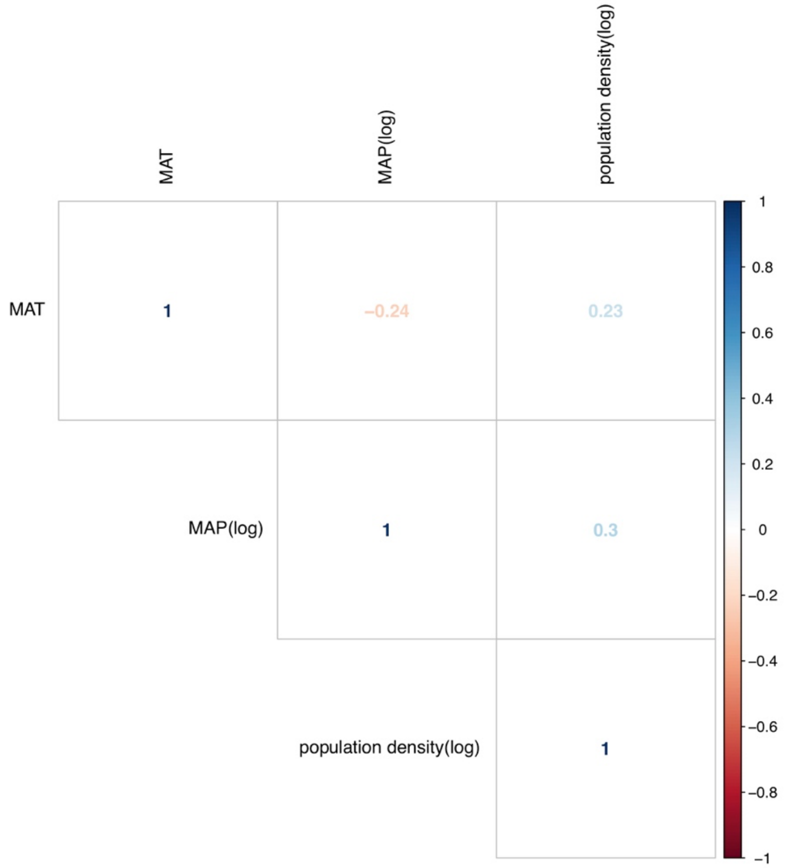

Fig. S3. Correlations among continuous predictor variables from the body mass dataset. All correlations are low.

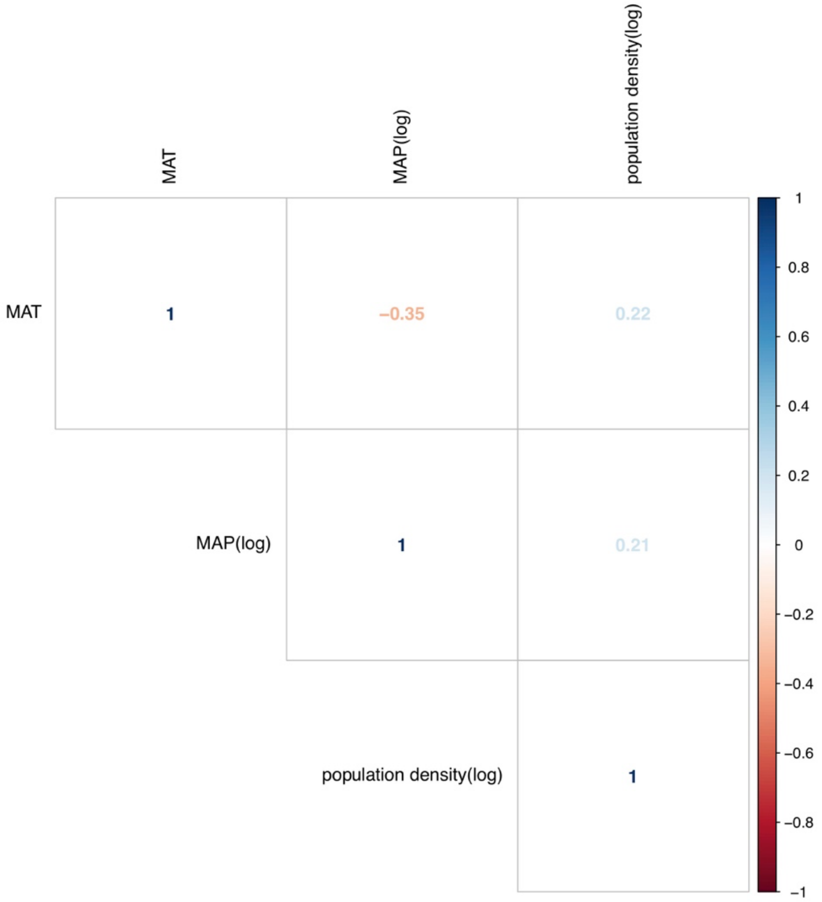

Fig. S4. Correlations among continuous predictor variables from the head-body length dataset. All correlations are low.

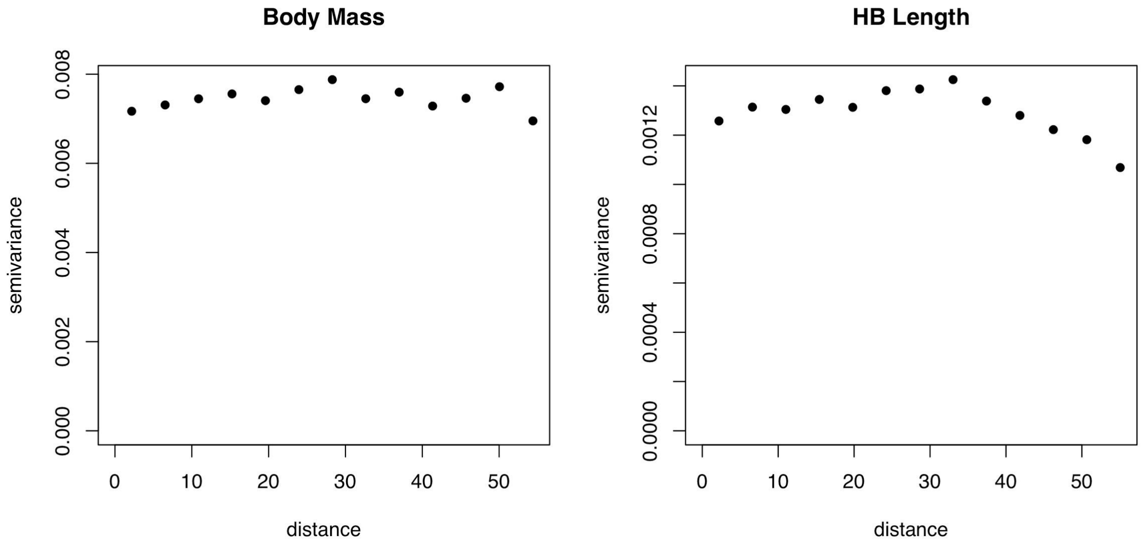

Fig. S5. Spatial autocorrelation residuals of the final body mass and head-body (HB) length models.

SUPPLEMENTAL TABLE

Table S1. Top body mass and head-body length LMM model results. Values represent estimates and standard errors. Bold effects are significant. Body mass LMM marginal $R^2 = 0.42$ and conditional $R^2 = 0.98$. Head-body length LMM marginal $R^2 = 0.51$ and conditional $R^2 = 0.98$.

Term	Body Mass	Head-body Length
(Intercept)	**2.607 ± 0.294**	**2.398 ± 0.102**
MAT	**-0.061 ± 0.004**	**-0.018 ± 0.001**
MAP	**-0.001 ± 0.000**	**-0.001 ± 0.000**
season:spring	**0.025 ± 0.001**	**0.008 ± 0.000**
Season:summer	**0.013 ± 0.001**	**0.002 ± 0.000**
Season:winter	**-0.007 ± 0.001**	0.000 ± 0.000
Sex:male	**0.002 ± 0.000**	
Population density	**0.008 ± 0.001**	**0.007 ± 0.001**
Hibernation:hibernator	0.234 ± 0.195	0.034 ± 0.067
Hibernation:none	**0.464 ± 0.163**	**0.147 ± 0.056**
Habitat buffering:none	0.365 ± 0.194	0.114 ± 0.066
Habitat buffering:obligate	**0.503 ± 0.193**	**0.132 ± 0.066**
Activity time:diurnal	0.170 ± 0.179	0.042 ± 0.061
Activity time:nocturnal	-0.027 ± 0.138	-0.048 ± 0.048
Small/large body size:small	**-1.809 ± 0.123**	**-0.579 ± 0.042**
MAT x small/large body size:small	**0.024 ± 0.003**	**0.005 ± 0.001**
Population density x small/large body size:small		**-0.003 ± 0.001**
MAT x population density	**-0.003 ± 0.000**	
MAT x hibernation:hibernator	**0.008 ± 0.002**	**0.007 ± 0.001**
MAT x hibernation:none	**0.019 ± 0.001**	**0.009 ± 0.001**
MAT x habitat buffering:none	**0.026 ± 0.002**	**0.007 ± 0.001**
MAT x habitat buffering:obligate	**0.016 ± 0.002**	**0.003 ± 0.001**
Population density x activity time:diurnal	**-0.015 ± 0.001**	**-0.006 ± 0.001**
Population density x diurnal/nocturnal:nocturnal	**-0.002 ± 0.001**	**-0.007 ± 0.000**

SUPPLEMENTAL REFERENCES

1. Myers, P., Espinosa, R., Parr, C.S., Jones, T., Hammond, G.S. & Dewey, T.A. (2015). The animal diversity web. See http://animaldiversity.ummz.umich.edu (accessed 22 August 2020).

2. Belk, M.C. & Smith, H.D. (1991). *Ammospermophilus leucurus*. Mammalian Species, 368, 1–8.

3. Carraway, L.N. & Verts, B.J. (1993). *Aplodontia rufa*. Mammalian Species, 431, 1–10.

4. Lovejoy, B.P. & Black, H.C. (1974). Growth and weight of the mountain beaver, *Aplodontia rufa pacifica*. Journal of Mammalogy, 55, 364–369.

5. Feldhamer, G.A., Thompson, B.C., Chapman, J.A. (2003). Wild mammals of North America: biology, management, and conservation. Baltimore, MD: Johns Hopkins University Press.

6. Bekoff, M. (1977). *Canis latrans*. Mammalian Species, 79, 1–9.

7. Mech, L.D. (1974). *Canis lupus*. Mammalian Species, 37, 1–6.

8. Paulson, D.D. (1988). *Chaetodipus baileyi*. Mammalian Species 297, 1–5.

9. Jameson, E.W., Jr., & H.J. Peeters. (2004). Mammals of California. University of California Press, Berkeley, USA.

10. Ziv, Y. & Smallwood, J.A. (2000). Gerbils and heteromyids – interspecific competition and the spatio-temporal niche. In Activity Patterns in Small Mammals: An Ecological Approach (eds. S. Halle and N. C. Stenseth), pp. 159–176. Ecological Studies, Springer Verlag.

11. Davidow-Henry, B.R., Jones, J.K. Jr., Hollander, R.R. (1989). *Cratogeomys castanops*. Mammalian Species, 338, 1–6.

12. Hedgal, P.L., Ward, A.L., Johnson, A.M. & Tietjen, H.P. (1965). Notes on the life history of the Mexican pocket gopher (*Cratogeomys castanops*). J Mammal., 46:334–335.

13. Hoogland, J.L. (1996). *Cynomys ludovicianus*. Mammalian Species, 535, 1–10.

14. McManus, J.J. (1974). *Didelphis virginiana*. Mammalian Species, 40, 1–6.

15. Price, M.V., Longland, W.S., Goldingay, R.L., (1991). Niche relationships of *Dipodomys agilis* and *D. stephensi*: two sympatric kangaroo rats of similar size. American Midland Naturalist, 126, 172–186.

16. Bleich, V.C., Price, M.V. (1995). Aggressive behavior of *Dipodomys stephensi*, an endangered species, and *Dipodomys agilis*, a sympatric congener. J Mammal., 76, 646–651.

17. Wolff, J.O., Sherman, P.W. (2007). Rodent Societies: An Ecological and Evolutionary Perspective. University of Chicago Press, Chicago, Illinois.

18. Garrison, T.E. & Best, T.L. (1990). *Dipodomys ordii*. Mammalian Species, 353, 1–10.

19. Kurta, A. & Baker, R.H. (1990). *Eptesicus fuscus*. Mammalian Species, 356, 1–10.

20. Connior, M.B. (2011). *Geomys bursarius*. Mammalian Species, 43, 104–117.

21. Streubel, D.P. & Fitzgerald, J.P. (1978). *Gulo gulo*. Mammalian Species, 103, 1–5.

22. Larivière, S. & Walton, L.R. (1998). *Lontra canadensis*. Mammalian Species, 587, 1–8.

23. Larivière, S. & Walton, L.R. (1997). *Lynx rufus*. Mammalian Species, 563, 1–8.

24. Frase, B.A. & Hoffmann, R.S. (1980). *Marmota flaviventris*. Mammalian Species, 135, 1–8.

25. Kwiecinski, G.G. (1998). *Marmota monax*. Mammalian Species, 591, 1–8.

26. Clark, T.W., Anderson, E., Douglas, C. & Strickland, M. (1987). *Martes americana*. Mammalian Species, 289, 1–8.

27. Cudworth, N.L. & Koprowski, J.L. (2010). *Microtus californicus* (Rodentia: Cricetidae). Mammalian Species, 42, 230–243.

28. Smolen, M.J. & Keller, B.L. (1987). *Microtus longicaudus*. Mammalian Species, 271, 1–7.

29. Sera, W.E. & Early, C.N. (2003). *Microtus montanus*. Mammalian Species, 716, 1–10.

30. Stalling, D.T. (1990). *Microtus ochrogaster*. Mammalian Species, 355, 1–9.

31. Carraway, L.N. & Verts, B.J. (1985). *Microtus oregoni*. Mammalian Species, 233, 1–6.

32. Reich, L.M. (1981). *Microtus pennsylvanicus*. Mammalian Species, 159, 1–8.

33. Smolen, M.J. (1981). *Microtus pinetorum*. Mammalian Species, 147, 1–7.

34. Cornely, J.E. & Verts, B.J. (1988). *Microtus townsendii*. Mammalian Species, 325, 1–9.

35. King, C.M. (1983). *Mustela erminea*. Mammalian Species, 195, 1–8.

36. Whitaker, J.O. Jr. & Wrigley, R.E. (1972). *Napaeozapus insignis*. Mammalian Species, 14, 1–6.

37. Macêdo, R.H. & Mares, M.A. (1988). *Neotoma albigula*. Mammalian Species, 310, 1–7.

38. Verts, B.J. & Carraway, L.N. (2002). *Neotoma lepida*. Mammalian Species, 699, 1–12.

39. Cornely, J.E. & Baker, R.J. (1986). *Neotoma mexicana*. Mammalian Species, 262, 1–7.

40. Braun, J.K. & Mares, M.A. (1989). *Neotoma micropus*. Mammalian Species, 330, 1–9.

41. Smith, A.T. & Weston, M.L. (1990). *Ochotona princeps*. Mammalian Species, 352, 1–8.

42. Smith, W.P. (1991). *Odocoileus virginianus*. Mammalian Species, 388, 1-13.

43. Willner, G.R., Feldhamer, G.A., Zueker, E.E. & Chapman, J.A. (1980). *Ondatra zibethicus*. Mammalian Species, 141, 1–8.

44. Best, T.L. & Skupski, M.P. (1994). *Perognathus flavus*. Mammalian Species, 471, 1–10.

45. Flake, L. & C.D. Jorgensen. (1969). Invasion of a "trapped out" southern Nevada habitat by *Perognathtis longimembris*. Great Basin Nat., 29, 143–149.

46. Bartholomew, G.A. & Cade, T.J. (1957). Temperature regulation, hibernation, and aestivation in the Little Pocket Mouse, *Perognathus longimembris*. Journal of Mammalogy, 38, 60–72.

47. US Fish and Wildlife Service (USFWS). (2010). Pacific pocket mouse (*Perognathus longimembris pacificus*) 5-Year Review: Summary and Evaluation. Carlsbad Fish and Wildlife Office: Service USFW.

48. Verts, B.J. & Kirkland, G.L. Jr. (1988). *Perognathus parvus*. Mammalian Species, 318, 1–8.

49. Johnson, D.W. & Armstrong, D.M. (1987). *Peromyscus crinitus*. Mammalian Species, 287, 1–8.

50. Lackey, J.A., Huckaby, D.G. & Ormiston, B.G. (1985). *Peromyscus leucopus*. Mammalian Species, 247, 1–10.

51. Hoffmeister, D.F. (1981). *Peromyscus truei*. Mammalian Species, 161, 1–5.

52. McAllister, J.A. & Hoffmann, R.S. (1988). *Phenacomys intermedius*. Mammalian Species, 305, 1–8.

53. Lotze, J.-H. & Anderson, S. (1979). *Procyon lotor*. Mammalian Species, 119, 1–8.

54. Spencer, S.R. & Cameron, G.N. (1982). *Reithrodontomys fulvescens*. Mammalian Species, 174, 1–7.

55. Webster, W.D. & Jones, J.K. Jr. (1982) *Reithrodontomys megalotis*. Mammalian Species, 167, 1–5.

56. Hartman, G.D. & Yates, T.L. (1985). *Scapanus orarius*. Mammalian Species, 253, 1–5.

57. Carraway, L.N. & Verts, B.J. (1994). *Sciurus griseus*. Mammalian Species, 474, 1–7.

58. Koprowski, J.L. (1994). *Sciurus niger*. Mammalian Species, 479, 1–9.

59. Gwinn, R.N., Palmer, G.H., Koprowski, J.L. (2011). *Sigmodon arizonae*. Mammalian Species, 883, 149–154.

60. Cameron, G.N., & Spencer, S.R. (1981). *Sigmodon hispidus*. Mammalian Species, 158, 1–9.

61. Whitaker, J.O. Jr. (2004). *Sorex cinereus*. Mammalian Species, 743, 1–9.

62. Carraway, L.N. (1985). *Sorex pacificus*. Mammalian Species, 231, 1–5.

63. Reid, F.A. (2006). A Field Guide to Mammals of North America North of Mexico. Houghton Mifflin, Boston.

64. George, S.B. (1989). *Sorex trowbridgii*. Mammalian Species, 337, 1–5.

65. Lomolino, M.V., Perault, D.R. (2007). Body size variation of mammals in a fragmented, temperate rainforest. Conserv. Biol., 21, 1059–1069.

66. Gillihan, S.W. & Foresman, K.R. (2004). *Sorex vagrans*. Mammalian Species, 744, 1–5.

67. Clothier, R.R. (1955). Contribution to the life history of *Sorex vagrans* in Montana. J. Mamm., 36, 214–221.

68. Chapman, J.A. & Willner, G.R. (1978). *Sylvilagus audubonii*. Mammalian Species, 106, 1–4.

69. Sutton, D.A. (1992). *Tamias amoenus*. Mammalian Species, 390, 1–8.

70. Best, T.L. (1993). *Tamias ruficaudus*. Mammalian Species, 452, 1–7.

71. Sutton, D.A. (1993). *Tamias townsendii*. Mammalian Species, 435, 1–6.

72. Long, C.A. (1973). *Taxidea taxus*. Mammalian Species, 26, 1–4.

73. Verts, B.J. & Carraway, L.N. (1999). *Thomomys talpoides*. Mammalian Species, 618, 1–11.

74. Fritzell, E.K. & Haroldson, K.J. (1982). *Urocyon cinereoargenteus*. Mammalian Species, 189, 1–8.

75. McGrew, J.C. (1979). *Vulpes macrotis*. Mammalian Species, 123, 1–6.

76. Larivière, S. & Pasitschniak-Arts, M. (1996). *Vulpes vulpes*. Mammalian Species, 537, 1–11.

77. Whitaker, J.O. Jr. (1972). *Zapus hudsonius*. Mammalian Species, 11, 1–7.

78. Hart, E.B., Belk, M.C., Jordan, E., Gonzalez, M.W. (2004). *Zapus princeps*. Mammalian Species, 749, 1–7.
