# Supplementary material for: Mammalian body size is determined by interactions between climate, urbanization, and ecological traits
Source: Commun Biol. 2021 Aug 16;4:972. doi: 10.1038/s42003-021-02505-3 (PMC8367959; doi:10.1038/s42003-021-02505-3)
Supplement: Supplementary file 3 — Description of Supplementary Files [file 42003_2021_2505_MOESM3_ESM.pdf]

Description of Additional Supplementary Files

File name: Supplementary Data 1

Description: Species trait information. Sample sizes are provided for each species in the body mass and head-body (HB) length datasets. Ecological traits include: hibernation (NONE = does not hibernate, DT = daily torpor, HIB = hibernator); hibernation binary (N = no, Y = yes); habitat buffer, three-state habitat buffering (O = obligate, F = facultative, N = does not buffer); activity time, binned mean body mass, and binned mean head-body (HB) length. Body mass, total length, tail length, and head-body (HB) length ranges extracted from the literature. R2 values are based on regressions of log10 head-body length and log10 body mass for each species. References are provided for ecological and morphological traits for each species in the Supplementary Information.
