# Supplementary material for: Mammalian body size is determined by interactions between climate, urbanization, and ecological traits
Source: Commun Biol. 2021 Aug 16;4:972. doi: 10.1038/s42003-021-02505-3 (PMC8367959; doi:10.1038/s42003-021-02505-3)
Supplement: Supplementary file 5 — Reporting Summary [file 42003_2021_2505_MOESM5_ESM.pdf]

nature research

Corresponding author(s): Maggie Hantak

Last updated by author(s): Jul 13, 2021

Reporting Summary

Nature Research wishes to improve the reproducibility of the work that we publish. This form provides structure for consistency and transparency in reporting. For further information on Nature Research policies, see our Editorial Policies and the Editorial Policy Checklist.

Statistics

For all statistical analyses, confirm that the following items are present in the figure legend, table legend, main text, or Methods section.

n/a	Confirmed	
☐	☒	The exact sample size (*n*) for each experimental group/condition, given as a discrete number and unit of measurement
☐	☒	A statement on whether measurements were taken from distinct samples or whether the same sample was measured repeatedly
☐	☒	The statistical test(s) used AND whether they are one- or two-sided<br>*Only common tests should be described solely by name; describe more complex techniques in the Methods section.*
☐	☒	A description of all covariates tested
☐	☒	A description of any assumptions or corrections, such as tests of normality and adjustment for multiple comparisons
☐	☒	A full description of the statistical parameters including central tendency (e.g. means) or other basic estimates (e.g. regression coefficient) AND variation (e.g. standard deviation) or associated estimates of uncertainty (e.g. confidence intervals)
☐	☒	For null hypothesis testing, the test statistic (e.g. F, t, r) with confidence intervals, effect sizes, degrees of freedom and P value noted<br>*Give P values as exact values whenever suitable.*
☐	☒	For Bayesian analysis, information on the choice of priors and Markov chain Monte Carlo settings
☐	☒	For hierarchical and complex designs, identification of the appropriate level for tests and full reporting of outcomes
☐	☒	Estimates of effect sizes (e.g. Cohen's d, Pearson's r), indicating how they were calculated

Our web collection on statistics for biologists contains articles on many of the points above.

Software and code

Policy information about availability of computer code

Data collection	All data was obtained from online data repositories and cleaned with R 3.6.1, R Core Team (2019).
Data analysis	All data analyses were done with R 3.6.1 statistical software. The main R code for this study is deposited on GitHib. https://github.com/mhantak/Mammal_spatial.git

For manuscripts utilizing custom algorithms or software that are central to the research but not yet described in published literature, software must be made available to editors and reviewers. We strongly encourage code deposition in a community repository (e.g. GitHub). See the Nature Research guidelines for submitting code & software for further information.

Data

Policy information about availability of data

All manuscripts must include a data availability statement. This statement should provide the following information, where applicable:

- Accession codes, unique identifiers, or web links for publicly available datasets
- A list of figures that have associated raw data
- A description of any restrictions on data availability

All data used in this study are available on GitHub. https://github.com/mhantak/Mammal_spatial.git

April 2020

Field-specific reporting

Please select the one below that is the best fit for your research. If you are not sure, read the appropriate sections before making your selection.

☐ Life sciences ☐ Behavioural & social sciences ☒ Ecological, evolutionary & environmental sciences

For a reference copy of the document with all sections, see nature.com/documents/nr-reporting-summary-flat.pdf

Ecological, evolutionary & environmental sciences study design

All studies must disclose on these points even when the disclosure is negative.

Study description	In this study we used a hierarchical modeling framework to test how climate and human population density (a proxy for urbanization) affect changes in mammal body size (body mass and head-body length). We also tested how species life history traits mediate changes in mammal body size by examining biologically relevant interactions between climate, urbanization, and traits.
Research sample	We obtained data from three repositories: VertNet (http://vertnet.org, Guralnick & Constable 2010); the National Ecological Observatory Network (NEON, https://www.neonscience.org/); and the North American Census of Small Mammals (NACSM, Calhoun 1948, 1949, 1950, 1951, 1956; Calhoun & Arata 1957a, 1957b, 1957c, 1957d). Human population density data were obtained from Fang and Jawitz (2018). Climate data were downloaded from PRISM Climate Group (PRISM Climate Group, 2020).
Sampling strategy	We used as much data as possible, but had a minimum filtering step of 100 records for body mass or length count per species.
Data collection	R. Guralnick obtained mammal body size data from VertNet, M. Hantak downloaded size data from NEON, and B. McLean manually digitized size data from NACSM reports. Climate and human population density data were extracted from online sources by D. Li.
Timing and spatial scale	We used mammal body size records that spanned between years 1940 to 2019. We used decade as a random effect in our models to account for temporal autocorrelation. The records used in this study span the conterminous USA.
Data exclusions	We excluded mammal species that had less than 100 body size records prior to additional filtering. We excluded migratory species because they experience a wide range of environmental conditions. In addition we manually excluded records that came from zoos or sanctuaries. Lastly, we filtered out juveniles because we were only interested in adult body size.
Reproducibility	All analyses were done using R statistical software and saved as an R script.
Randomization	N/A, we did not perform experiments in this study.
Blinding	N/A, we did not perform experiments in this study.

Did the study involve field work? ☐ Yes ☒ No

Reporting for specific materials, systems and methods

We require information from authors about some types of materials, experimental systems and methods used in many studies. Here, indicate whether each material, system or method listed is relevant to your study. If you are not sure if a list item applies to your research, read the appropriate section before selecting a response.

Materials & experimental systems

n/a	Involved in the study	
☒	☐	Antibodies
☒	☐	Eukaryotic cell lines
☒	☐	Palaeontology and archaeology
☒	☐	Animals and other organisms
☒	☐	Human research participants
☒	☐	Clinical data
☒	☐	Dual use research of concern

Methods

n/a	Involved in the study	
☒	☐	ChIP-seq
☒	☐	Flow cytometry
☒	☐	MRI-based neuroimaging
