# Supplementary material for: Mammalian body size is determined by interactions between climate, urbanization, and ecological traits
Source: Commun Biol. 2021 Aug 16;4:972. doi: 10.1038/s42003-021-02505-3 (PMC8367959; doi:10.1038/s42003-021-02505-3)
Supplement: Supplementary file 1 — Transparent Peer Review File [file 42003_2021_2505_MOESM1_ESM.pdf]

Reviewers' comments:

Reviewer #1 (Remarks to the Author):

Summary Comments
The manuscript, "Mammalian body size is determined by interactions between climate, urbanization, and life history traits", presents an individual-level analysis of mammalian body size variation across the United States in relation to climate and human population density. The scope of this work is impressive and the scale of data unmatched, particularly for specimen-associated, individual-level measurements of body size and geolocations. This dataset will no doubt set a standard moving forward for studies of this type. The incorporation of extrinsic (climate, urbanization) and intrinsic drivers (traits) provides a strong and comprehensive analysis framework in general. I believe this paper will ultimately make an excellent contribution and be of interest to a very wide audience, but I have several major concerns and recommend possible solutions to improve the robustness of the analyses and clarity of the writing. Key among my concerns are: 1) using climate data from the year of observation may not represent the time period contributing most to an individual's growth, 2) the lack of explanation about how species were assigned to the "Habitat buffering" trait and the lumping of obligate hibernators together with species that facultatively use torpor for the "hibernation" trait, which makes their interpretations fraught, and 3) the reliance on models that do not incorporate phylogeny may mislead conclusions because they ignore an important source of variance in the data. I include more detailed explanation of these below, along with other minor concerns about what is overall a very well-done, interesting, and novel study.

Specific Comments
ABSTRACT
1. In the Abstract, and throughout the manuscript, I would urge you to be more careful with word choice to clarify that this analysis addresses spatial variation and not temporal variation (which is indeed stated several times, but seemed at odds with the language used at times). The results have obvious implications for climate and land cover change (i.e. temporal change) and many of the studies cited do focus on change over time, but the scope of the present analysis needs to be very clear and consistent. For example, the use of "warming temperatures" (e.g. Lines 35, 390) connotes climate change to me, when really you mean in locations that had warmer temperatures. Another frequent source of confusion was your use of the word "change" (eg, Lines 29, 31, 402), which, in most cases, I think may be better as "variation" or "differences" or something similar. Until I reached the Methods, the language used had me expecting explicit analyses of intraspecific change over time in body size as climate and land cover (~population density) has changed, whereas time was strictly treated as a nuisance variable and variation over space (and associated environmental gradients) is the sole focus. The Abstract and Discussion seem to be most affected by this. I'd recommend sticking to the types of wording used in the paragraph starting on Line 103 ("In this study, we compiled multiple….") which is very clear and effective.

INTRODUCTION
2. Line 70 is missing a word (in?): "…but limited support endotherms…"
3. Line 83, I generally don't consider body size a life history trait. Indeed, you separate them below (Line 88-89). This term gets thrown around so loosely in the literature that it is a pet peeve of mine. As a solution, simply make it "traits" here?
4. Line 128-129. There is some evidence that obligate hibernators may actually obtain larger body sizes under warming conditions if the growing season is extended, allowing for longer periods of resource consumption and allocation to growth. Thus, they may not be "less sensitive" but rather show responses to temperature that are counter to typical Bergmann expectations. The most relevant example is Eastman, Lindsey M., et al. "Size increase in high elevation ground squirrels over the last century." Global Change Biology 18.5 (2012): 1499-1508. A review by Yom-Tov and Geffen ("Recent spatial and temporal changes in body size of terrestrial vertebrates: probable causes and pitfalls." Biological Reviews 86.2 (2011): 531-541) contains other relevant discussion and citations of the link between food supply and body size that you may deem useful regarding these predictions and hypotheses. See my additional comments on this in comment #11, below.
5. Line 130. Here you refer to this trait as "diurnality", but elsewhere (eg Fig 3 caption) you use "activity time", which is preferable.

METHODS
6. Line 157 – Here you report the minimum number of records required for a species to be used in the analysis, but how variable are sample sizes among species (and/or trait categories)? It seems like very uneven sampling could bias some of the trait-based results and interpretations. Including the sample sizes per species in Supplementary Materials would be helpful – ideally along with the trait information if possible (e.g., Figure S2) but not necessarily.
7. Population density – I agree that population density is generally a reasonable proxy for urbanization, but I worry that the use of population density may dampen trends related to resource hypotheses. Rural areas with low population density but high agricultural crop cover also provide substantial food resources to many mammals. This caveat should at least be addressed in the Discussion.
8. Climate data – Using the observation year of specimens to obtain climate data accounts well for any climate changes at a location over the study period broadly, but single-year means may not best quantify the conditions of the true time period of consequence because (1) environmental resource levels and thus body condition (e.g. mass) can lag behind weather events/climate conditions and (2) mammals exhibit determinate growth with asymptotic adult body sizes reached relatively early in an individual's life, making the conditions during this early stage the critical growth period of an individual that leads to its ultimate mass or HB length. Thus, simply using the year of observation may introduce mismatches and additional error that can be avoided. As an extreme example, annual climate means for a given year would have little relevance to the mass or HB length of a specimen captured on January 2nd of that year. Or, consider a 5 year old animal which grew to its mature size in its first year of life, during which the MAT and MAP could have been quite different (e.g. during a drought event). Averaging climate across multiple years, including the year of observation and one to several years prior, is arguably more appropriate and would reduce the impact of anomalous years. However, the difficulty with finding a solution to this with this data set is that 1) knowing the true age of individuals is impossible for most observations and 2) the study species differ dramatically in average life span and growth rate, so determining the correct window to average over would be very challenging. In lieu of averaging across a larger time window, is it possible to calculate climate means for the 12 months prior to and including the month of capture rather than simply the calendar year of observation? That way at least no climate data would be used that an individual didn't experience. This is a unique challenge of using individual-level body size data on this scale.
For references re: asymptotic growth in mammals see Creighton, G. Ken, and Richard E. Strauss. "Comparative patterns of growth and development in cricetine rodents and the evolution of ontogeny." Evolution 40.1 (1986): 94-106. and West, Geoffrey B., James H. Brown, and Brian J. Enquist. "A general model for ontogenetic growth." Nature 413.6856 (2001): 628-631.
9. Spatial regions – Why wasn't the Eastern Temperate Forest split up the way other large ecoregions were?
10. Mammal traits – Many of the major results and conclusions of the analysis rest on the species traits but without more description of exactly how the habitat buffering and hibernation trait states were defined and assigned to species, it is difficult to judge their robustness. These two traits are not simple to define and can be quite subjective, so more justification and explanation are needed for both, but I am most concerned about habitat buffering assignments here. Slightly more information is provided in the caption of Fig S2, but it also is rather vague and doesn't help explain what appear to be surprising species' assignments. For example, what explains why only 1 pocket mouse (Chaetodipus baileyi) is "buffered" while the rest are not when their nesting and foraging habits are all very similar? Similarly, the distinction between some ground squirrels and chipmunks seems arbitrary (or purely phylogenetic) as all basically nest in burrows but forage above ground, including some in desert environments during the day. I was also surprised to see volant species as "buffered" given that metabolic demands of flight are often cited as a reason bats are more sensitive to temperature. And how were aquatic/semi-aquatic species handled? It appears the semi-aquatic rodents were considered "buffered" but not semi-aquatic carnivores (Lontra canadensis and Neovision vison). For all of these, simply citing your reasoning/methods in a bit more detail may help explain them: for example, did you rely on specific citations? Identification of keywords in species accounts? One primary source then others to fill gaps? Genus-level descriptions? Was other information besides activity location used?. Without greater explanation as to how these categories were assigned, I have pretty strong concerns about this trait's results.
11. For hibernation ability (related to my comment from Line 128-129), the way you've done it seems adequate for a broad look at the trait, but I worry that by lumping together species that use

daily torpor with annual obligate hibernators, you may be conflating two different strategies and therefore potentially different responses. At the very least, sensitivity analyses such as those for body size bins seem warranted, in which you further split these sub-groups of dormancy. Just among rodents for example, the body size response to temperature of some of the ground squirrels, jumping mice, and small heteromyids (e.g. genus Perognathus) that are completely inactive (or nearly so) for several months are likely affected by differences in their resource acquisition due to the length of the growing season during which they are active (See the Eastman et al paper, above). In contrast, species that go into daily torpor are doing so more strictly to evade challenging temperatures. (See Geiser, Fritz, and Thomas Ruf. "Hibernation versus daily torpor in mammals and birds: physiological variables and classification of torpor patterns." Physiological Zoology 68.6 (1995): 935-966.) Aestivation versus hibernation is another possible discrepancy in the broad trait of dormancy, though the relatively low number of mammals that aestivate makes it less likely to change your results I would guess. Note that this idea comes up in the Discussion as well (Line 365).
12. Line 251 – The overall results may be largely concordant, but models not incorporating phylogeny ignore an important and known source of variance in the data (phylogenetic signal) and thus are invalid for drawing conclusions. It is therefore more appropriate to report, interpret, and discuss the phylogenetic corrected models in the main text and put the "traditional" or non-phylogenetic contrast methods in the Supplemental. Moreover, by accounting for the variance due to phylogeny, the PGLMM model results cut more directly to your main objective: isolating the ecological patterns (above and beyond the effect of phylogeny and other non-focal variables, such as sex, season, etc.). For this dataset especially, which span deeply divergent lineages, accounting for phylogenetic relationships seems critical. Furthermore, all of the traits in question have often been shown to have moderate to strong phylogenetic signal across mammals or within some of the orders or families studied, so phylogeny cannot simply be ignored.

RESULTS
13. Table 1. I find it very difficult to easily compare the results for mass vs HBL as currently formatted. Could A and B be aligned next to each other? Also, the β symbol should be included in the Table header to more clearly link the text to the table. Same comments for Table S3.
14. In all Figures, it should be clear that the temperature values are on a standardized scale, not raw temperatures. Unless the US is just much, much colder than I realize. :)

DISCUSSION
15. Line 307-308 – I think your results present strong and compelling evidence for the effect of population density, but it's not clear on what evidence you make the claim that population density is more tightly linked to body size than temperature. Don't your results indicate that it depend on the metric of body size? If you are basing this on the fact that the main effect of MAT is only significant for 1 metric (mass), I would note that both are significant in your PGLMM model, which is the valid set of results to interpret. And the magnitude of the main effects is still opposite for HB versus mass. A version of this statement which appears in the abstract should also be revised unless there is other evidence presented to support the claim.
16. Lines 371-374 – Excellent point. There are a couple good examples out there you may choose to cite: The Eastman et al 2012 paper looks at exactly that, as well as a brand new paper that may be nicely complementary to many of the ideas you present: E. A. Riddell, K. J. Iknayan, L. Hargrove, S. Tremor, J. L. Patton, R. Ramirez, B. O. Wolf, S. R. Beissinger. Exposure to climate change drives stability or collapse of desert mammal and bird communities. Science. 05 Feb 2021 : 633-636.

Reviewer #2 (Remarks to the Author):

In their manuscript, Mammalian body size is determined by interactions between climate, urbanization and life history traitis, Hantak et al. set out to test how climate, and urbanization interact with species traits to determine body size in a over 100 mammal species. They conclude that urbanization is associated with larger size, suggesting resource availability is a stronger determinant of size than temperature. This effect is mediated by life history and size itself, with larger species and those with thermal buffering life history strategies being more sensitive to

temperature change. This is a well laid out manuscript, addressing an interesting set of questions that are clearly outlined and compellingly justified. I have several fairly major concerns, but if the authors are able to address them, I think this could be an exciting and important contribution. I also have some minor suggestions.

My main concerns are:

1) There is limited consideration of the mechanism by which temperature-driven size reduction may be happening. This has led to limited consideration of what temperature variable matters. Is the mean annual temperature of the cell where a specimen was collected really the temperature that would determine body size? Shouldn't it be the temperature in the location and at the time that reproduction occurred? I think this deserves much greater consideration both conceptually and empirically (e.g. through sensitivity analyses).

2) Given urban heat island effects, it seems like temperature and human pop density should be highly correlated. Given the complexity of the model, if this is the case, it seems like it might be hard to interpret the directionality of the relationships recovered in the model.

3) The treatment of time and size in the model seem unnecessarily imprecise. Why use a random effect for decade when you could include year as a fixed effect? Why not use mean body size for a species as a predictor rather than binning the data?

4) I am of the opinion that this paper should not be published without explicit acknowledgement of each of the museums that house the specimens used; I recommend acknowledging the institutions at the least, and the curators-in-charge and collections managers from mammalogy if possible. Given the scope of the dataset, this could be done in supplemental materials.

5) Assuming the effect sizes are comparable, the effect of MAT is higher than the effect of population density; this seems at odds with the framing of the paper, that urbanization is more tightly linked to size than temperature. Apologies if I've misinterpreted this somehow.

Minor concerns:

Line 145: Does total body length include tail? Is tail thermoregulatory, and could it be increasing as a result of Allen's Rule? Rather than reference Guralnick (2016) here without any detail; could you include enough so that people generally know what you did without being familiar with the reference? It seems like – given the later text – tail is excluded; this would be worth clarifying throughout.

Line 149 – How did you determine there were no systematic biases?

Why was population density log transformed for the modeling?

Why bin body size (Line 219) instead of using average size per species as a predictor?

Why not include Year as a fixed effect rather than decade as a random effect?

Why present the LMM results rather than the PGLMM results? I imagine the dredging is not possible with PGLMM, so it makes sense to do it with the LMMs, but once you have the best model, it seems like it makes sense to account for phylogeny.

I think you should present the correlations among the predictors as a supplemental table.

Typos in the Table 1 legend – (A) HB length.

The Introduction is light on references – see for example, the beginning of the paragraph that starts on Line 86.

Reviewer #3 (Remarks to the Author):

In this paper, Hantak and colleagues investigate variation in mammalian body size across the United States in relation to both climate variables and human population density (proxy for urbanization). The authors also investigate how traits such as diel activity patterns, hibernation

ability, and ability to “buffer” their habitat (e.g., via burrowing) influence these patterns in body size. The authors compiled a very large dataset of mammals and explored these relationships using linear mixed models. They found that, surprisingly, urbanization has a larger effect on mammalian body size than temperature and that mammals tend to be larger in urban environments. The authors also find that different life history traits also modulate these patterns, and they discuss implications for the dual effects of climate change and ongoing urbanization on mammal populations in the future.

I enjoyed this paper and think that the methods the authors used were appropriate to explore the questions proposed. The results are novel and should be of broad interest to conservationists, climate change researchers, mammalogists, and ecologists. I only had a few minor critiques, but these are mostly related to reorganizing certain sections or justifying certain methodological decisions.

(1) The authors should consider reorganizing the Introduction. The Abstract and Discussion are very well organized and have a logical flow that I was able to follow and anticipate the forthcoming section. In contrast, the Introduction felt a little jumbled and I struggled to follow from one point to the next. I offer specific suggestions in the comments-by-line below. In general, clearly setting up the major variables explored in this study and what the rationale was for investigating them will greatly improve the paper.
(2) Somewhat related to the Introduction, I found certain definitions/terminology to be used inconsistently in the paper or to not be clearly defined. For example, “life history traits”, “buffering traits”, “thermal tolerance traits”, or simply “traits” are often used interchangeably, or at least without sufficient definition of how they differ. I’ve left specific comments below, but in particular the idea of “buffering” and how one defines a “buffering” vs. “non-buffering” mammal would be helpful.
(3) In the figures/tables a more thorough description of the data being presented would be very helpful, especially for future readers who may be skimming through. Related to that, the comparison between Table 1 and Supplemental Table S3 would be much improved if the same format/columns were used for both (or if that is impossible some explanation as to why).

Below, I list my specific comments/suggestions. In general, I think the paper is well-written, well-conceived, and interpreted appropriately. In particular, I found the Discussion to be very well-written and concise. All of my comments should be considered minor, and I look forward to seeing this paper published in Communications Biology in the near future.

Sincerely,

Lucas N. Weaver

Comments by Line

Introduction

45: “understanding drivers of body size” sounds a bit odd, consider changing to “understanding drivers of body size variation” or something comparable.

45–46: Consider adding some relevant citations to make your point that understanding the drivers of body size variation has been a central goal of ecology over the last half century.

47: Most readers will no doubt be familiar with “Bergmann’s Rule,” but it would be worthwhile to briefly summarize his findings before adding the caveat statement, “…although with much debate…”

54: “While” is a time word, e.g., “While I was at the store my friend prepared dinner.” Consider changing to “Whereas.”

69: The authors state that heat island effects have been observed in “various animal taxa” but the

only reference provided is for insects. Consider adding additional references to make this point, or specify that the pattern is most readily observed in insects.

70: "…but limited support endotherms" is an incomplete sentence fragment. Consider revising to something like, "…but there is little support for heat island effects on endotherms."

70–71: The sentence that begins with, "Little attention…" seems out of place given the preceding sentence, and I am not sure it is necessary. The next point the authors make regarding the previous study on deer mice follows well without this intervening statement. Consider removing or making a segue between the two points.

76: Here the authors use a comma after "i.e." but elsewhere in the manuscript "e.g." and "i.e." are not followed by a comma. Consider keeping this consistent throughout.

76: The use of "Second" to begin the next portion of this paragraph implies there was a "first" but as constructed in is unclear what the "first" point was. I assume it is the observation that head-body length decreased but body mass did not in urbanized environments? The authors might consider reorganizing this paragraph to make their main points clearer. For example, a new paragraph could be started after the "limited support for endotherms" statement, then the authors could begin with a topic sentence outlining the main points they'll make in the paragraph. Something like, "Previous studies have hypothesized that urbanized environments may influence mammalian body size by (1) selecting for individuals that are able to avoid detection (i.e., crypsis), (2) providing greater food and water resources (i.e., resource rule), and (3) favoring changes in life history traits. First…etc." As currently written, it does not become clear that the authors are introducing hypotheses and predictions they aim to test in this study until the very end of the paragraph.

86–89: This first sentence is a hard to follow. Consider revising in a similar way as suggested in my previous comment. I agree that mammals are a good system for investigating the questions of interest to this study, but the reasons why can be more clearly outline in a topic sentence, then expanded upon in the meat of the paragraph, then re-emphasized at the end. For example, "Mammals represent a good study system for investigating how urbanization affects body size and life history traits because mammals are ecologically diverse, exhibit a wide range of body sizes, have diverse life history strategies, and are well-represented in biodiversity datasets." Or something like that.

89–91: Consider adding some citations to support the statement that "Mammals have evolved to fill…etc." To most readers this is probably obvious, but a few references would be useful to guide folks who might be less familiar with mammalian diversity/ecology. Even if it is a broad reference text like Walker's Mammals of the World (Nowak, RM, 1999, Johns Hopkins University Press).

91–92: Consider providing a reference(s) for the statement, "These traits are expected to strongly mediate…etc." If there are no previous studies that support this point, consider revising to say something like, "We expect that these traits strongly mediate….etc."

96–97: I'm not sure the preceding statement explains why habitat buffering might lessen the effect of urbanization on mammalian body size. This also seems a bit out of place in a paragraph about why mammals make a good study system. Consider either moving these thoughts into the preceding paragraph, or making it clear why mammals have better "buffering" capabilities compared to other organisms and why this might dampen the effects of urbanization on body size.

103–105: Consider mentioning from where this dataset was derived (e.g., compilation from primary literature) and also citing Supplemental Table S1 since it provides the references for each species. This is an impressive dataset!

105–107: Consider rewriting the sentence, "Our overarching question…etc." As currently written, it is a bit convoluted. For example, the explanation of what you mean by "human population density" could be put in the next sentence instead of placed in the middle of what is an important statement of your primary goal(s).

103–123: This is a great paragraph outlining the questions/hypotheses you'll be exploring in this paper. I think reorganizing the preceding paragraphs slightly as I suggested above will help guide the reader more naturally to these ideas.

124–128: Is there a reason the authors chose to refer to traits such as fossoriality, the ability to hibernate, and diel activity pattern as life history traits rather than ecological traits? Typically, I think of life history traits as being related to development and reproduction (e.g., growth rate, reproductive timing) rather than the way in which an animal interacts with its environment. This is not a major issue, and perhaps is a more common practice than I am aware of, but the authors might consider explaining/justifying their choice to refer to these as "life history traits".

Methods

195–197: The authors might consider clarifying why human population density, compiled from a source with 1x1 km resolution chose to aggregate into 10x10 km bins. Further, they might consider explaining what they mean by "appended to each record." I assume that the records are the mammalian occurrence records and that the reason for aggregating the more precise human population data into larger bins is due to the lower spatial resolution of the mammalian occurrence data, but this could be more clearly spelled out here.

198–199: Does the discrepancy in climate data resolution (4 km) and human population density resolution (10 km) pose any problems for downstream analyses?

215–216: Were your life history traits determined from the same sources listed at the beginning of the Methods section? If so, consider mentioning that here, and if not consider adding a brief statement of where these data came from.

217: For habitat buffering the authors present the binary "fossorial vs. terrestrial" as an example. The authors should consider listing all of the habitat buffering traits they used here, or at least point the reader to where they could find them (I may have missed something, but can't find it listed anywhere). Also, does "terrestrial" refer to all non-burrowing mammals (e.g., arboreal)? The authors should consider clarifying what these bins refer to, keeping in mind that many mammals that are considered "terrestrial" can also burrow (e.g., rats). Issues like these are unavoidable in large datasets like those used in this paper, but some appropriate caveats should be given nonetheless.

Results

265: The use of "Traits" to start this paragraph is referring to what the authors consider "life history traits," correct? If so, consider specifying; traits can mean anything in this context (e.g., body mass is a "trait").

267–269: Doesn't this sentence contradict the previous statement? Hibernation was one of the traits considered to be a habitat "buffer" correct? If there is a substantial difference between hibernators and other "buffer" types (e.g., burrowing) that should be noted? In general with this section, the authors should consider being precise and consistent in their terminology so as to avoid confusion. This issue persists in the next sentence about diurnal vs. nocturnal species, which I took to represent another form of "buffering".

Discussion

295–305: This paragraph is a great summary of the rationale behind the paper. Following a structure such as this would be useful in the Introduction; the authors might consider using this as a guide for how to more clearly set up the study early on.

Figures/Tables

Fig. 1: Great figure summarizing the spatial coverage and abundance of your data. Consider

defining the abbreviation HB at the beginning of the figure caption rather than at the end.

Fig. 2: Consider adding to this figure caption an explanation of how this plot was generated.

Table 1: Consider explaining in the caption where the data for different columns were derived from (such that someone skimming the paper could get a sense of your analyses).

Supplemental Information

Fig. S1: In the Introduction the authors mention that differences in areas sampled could influence your results (e.g., depauperate sampling of the southeast) but this point is never brought up again, or perhaps I missed it. Since you have this figure you might consider referring to it and adding some discussion about sampling gaps, perhaps in the Methods.

Fig. S2: In this caption is the first reference I've seen for how you've defined your "habitat buffering mammals" beyond the "fossorial vs. terrestrial" example. These should be more clearly defined and justified in the Methods. That fossorial and subterranean taxa can "buffer" their environments through burrowing makes sense to me, but I would not consider volant taxa (i.e., bats) to buffer their habitats through flying. I'm sure the authors have some rationale for making this assignment, but I am not familiar with it and I imagine the same would go for other readers.

Table S3: Is there a reason why the data reported for the PGLMM results are in a different format from the table in the primary text? This table is missing the standard error and p-value columns present in the other table. It would be helpful if the reader could directly compare the two results to see what effect phylogeny has on the modeling results. The congruence between the two models is pretty striking, especially given the dominance of rodents in the sample (which is to be expected), so I think providing that direct comparison would make your case even stronger.

Referee expertise:

Referee #1: Ecology, Biogeography, Systematics of Mammals

Referee #2: Evolutionary Ecology, Conservation, Macroevolution

Referee #3: Mammalian Evolution

Reviewers' comments:

Reviewer #1 (Remarks to the Author):

Summary Comments

The manuscript, "Mammalian body size is determined by interactions between climate, urbanization, and life history traits", presents an individual-level analysis of mammalian body size variation across the United States in relation to climate and human population density. The scope of this work is impressive and the scale of data unmatched, particularly for specimen-associated, individual-level measurements of body size and geolocations. This dataset will no doubt set a standard moving forward for studies of this type. The incorporation of extrinsic (climate, urbanization) and intrinsic drivers (traits) provides a strong and comprehensive analysis framework in general. I believe this paper will ultimately make an excellent contribution and be of interest to a very wide audience, but I have several major concerns and recommend possible solutions to improve the robustness of the analyses and clarity of the writing. Key among my concerns are: 1) using climate data from the year of observation may not represent the time period contributing most to an individual's growth, 2) the lack of explanation about how species were assigned to the "Habitat buffering" trait and the lumping of obligate hibernators together with species that facultatively use torpor for the "hibernation" trait, which makes their interpretations fraught, and 3) the reliance on models that do not incorporate phylogeny may mislead conclusions because they ignore an important source of variance in the data. I include more detailed explanation of these below, along with other minor concerns about what is overall a very well-done, interesting, and novel study.

We appreciate the interest and detailed constructive feedback on our manuscript. We took all reviewer comments seriously and have made several major adjustments to the manuscript, which we explain in detail below. Overall, we feel that suggested revisions have greatly improved the quality of this manuscript.

Specific comments

ABSTRACT

1. In the Abstract, and throughout the manuscript, I would urge you to be more careful with word choice to clarify that this analysis addresses spatial variation and not temporal variation (which is indeed stated several times, but seemed at odds with the language used at times). The results have obvious implications for climate and land cover change (i.e. temporal change) and many of the studies cited do focus on change over time, but the scope of the present analysis needs to be very clear and consistent. For example, the use of "warming temperatures" (e.g. Lines 35, 390) connotes climate change to me, when really you mean in locations that had warmer temperatures. Another frequent source of confusion was your use of the word "change" (eg, Lines 29, 31, 402), which, in most cases, I think may be better as "variation" or "differences" or something similar. Until I reached the Methods, the language used had me expecting explicit analyses of intraspecific change over time in body size as climate and land cover (~population density) has changed, whereas time was strictly treated as a nuisance variable and variation over space (and associated environmental gradients) is the sole focus. The Abstract and Discussion seem to be most affected by this. I'd recommend sticking to the types of wording used in the paragraph starting on Line

103 (“In this study, we compiled multiple….”) which is very clear and effective.

This is an important point. We updated the text throughout the manuscript to indicate that we were not focused on temporal trends, but spatial body size trends.

INTRODUCTION

2. Line 70 is missing a word (in?): “…but limited support endotherms…”

Yes, we were missing the word ‘in’. Now fixed.

3. Line 83, I generally don’t consider body size a life history trait. Indeed, you separate them below (Line 88-89). This term gets thrown around so loosely in the literature that it is a pet peeve of mine. As a solution, simply make it “traits” here?

Updated to “traits”.

4. Line 128-129. There is some evidence that obligate hibernators may actually obtain larger body sizes under warming conditions if the growing season is extended, allowing for longer periods of resource consumption and allocation to growth. Thus, they may not be “less sensitive” but rather show responses to temperature that are counter to typical Bergmann expectations. The most relevant example is Eastman, Lindsey M., et al. "Size increase in high elevation ground squirrels over the last century." Global Change Biology 18.5 (2012): 1499-1508. A review by Yom-Tov and Geffen ("Recent spatial and temporal changes in body size of terrestrial vertebrates: probable causes and pitfalls." Biological Reviews 86.2 (2011): 531-541) contains other relevant discussion and citations of the link between food supply and body size that you may deem useful regarding these predictions and hypotheses. See my additional comments on this in comment #11, below.

This is a good point. We added in this citation (Eastman et al.) as a caveat to our expectations in the introduction. In addition, we used this citation to expand the discussion of our findings.

The Yom-Tov and Geffen citation is an important one that was overlooked in the initially submitted manuscript. We now refer to this citation a few times throughout the manuscript.

5. Line 130. Here you refer to this trait as “diurnality”, but elsewhere (eg Fig 3 caption) you use “activity time”, which is preferable.

Agreed. We updated the terminology to ‘activity time’.

METHODS

6. Line 157 – Here you report the minimum number of records required for a species to be used in the analysis, but how variable are sample sizes among species (and/or trait categories)? It seems like very uneven sampling could bias some of the trait-based results and interpretations. Including the sample sizes per species in Supplementary Materials would be helpful – ideally along with the trait information if possible (e.g., Figure S2) but not necessarily.

We now report species sample size for both body mass models and head-body length models in Supplemental Table 1

7. Population density – I agree that population density is generally a reasonable proxy for urbanization, but I worry that the use of population density may dampen trends related to resource hypotheses. Rural

areas with low population density but high agricultural crop cover also provide substantial food resources to many mammals. This caveat should at least be addressed in the Discussion.

We agree and have added in this potential caveat to the discussion. Lines 359-361.

8. Climate data – Using the observation year of specimens to obtain climate data accounts well for any climate changes at a location over the study period broadly, but single-year means may not best quantify the conditions of the true time period of consequence because (1) environmental resource levels and thus body condition (e.g. mass) can lag behind weather events/climate conditions and (2) mammals exhibit determinate growth with asymptotic adult body sizes reached relatively early in an individual's life, making the conditions during this early stage the critical growth period of an individual that leads to its ultimate mass or HB length. Thus, simply using the year of observation may introduce mismatches and additional error that can be avoided. As an extreme example, annual climate means for a given year would have little relevance to the mass or HB length of a specimen captured on January 2nd of that year. Or, consider a 5 year old animal which grew to its mature size in its first year of life, during which the MAT and MAP could have been quite different (e.g. during a drought event). Averaging climate across multiple years, including the year of observation and one to several years prior, is arguably more appropriate and would reduce the impact of anomalous years. However, the difficulty with finding a solution to this with this data set is that 1) knowing the true age of individuals is impossible for most observations and 2) the study species differ dramatically in average life span and growth rate, so determining the correct window to average over would be very challenging. In lieu of averaging across a larger time window, is it possible to calculate climate means for the 12 months prior to and including the month of capture rather than simply the calendar year of observation? That way at least no climate data would be used that an individual didn't experience. This is a unique challenge of using individual-level body size data on this scale.
For references re: asymptotic growth in mammals see Creighton, G. Ken, and Richard E. Strauss. "Comparative patterns of growth and development in cricetine rodents and the evolution of ontogeny." Evolution 40.1 (1986): 94-106. and West, Geoffrey B., James H. Brown, and Brian J. Enquist. "A general model for ontogenetic growth." Nature 413.6856 (2001): 628-631.

We agree with the reviewer that there are complexities in modeling variation in body size with only using the sample year. To examine potential modeling caveats listed by the reviewer, we, in addition to running models with the year of sample collection as was done originally, ran models with mean temperature and precipitation averaged across 2 years (collection year + 1 year before) and 5 years (collection year + 4 years before). Our results were consistent across these different combinations of temperature and precipitation averages. We added text to explain this workflow, which we agree improves the stability of our modeling approach in the face of uncertainty inherent in our dataset that does not contain detailed information on individual age. Lines 238-241.

9. Spatial regions – Why wasn't the Eastern Temperate Forest split up the way other large ecoregions were?

The ecoregions we split into southern and northern regions have a larger latitudinal extent than the Eastern Temperate Forest ecoregion, which impart higher climatic and ecological provinciality as compared to the latter. We do recognize that there is some arbitrariness to any such regionalizations, no matter how they are defined. We have therefore chosen not to split the Eastern Temperate ecoregion in our revised manuscript, especially since regional variation in our original models were not particularly strong compared to other factors e.g. species.

10. Mammal traits – Many of the major results and conclusions of the analysis rest on the species traits but without more description of exactly how the habitat buffering and hibernation trait states were defined

and assigned to species, it is difficult to judge their robustness. These two traits are not simple to define and can be quite subjective, so more justification and explanation are needed for both, but I am most concerned about habitat buffering assignments here. Slightly more information is provided in the caption of Fig S2, but it also is rather vague and doesn't help explain what appear to be surprising species' assignments. For example, what explains why only 1 pocket mouse (Chaetodipus baileyi) is "buffered" while the rest are not when their nesting and foraging habits are all very similar? Similarly, the distinction between some ground squirrels and chipmunks seems arbitrary (or purely phylogenetic) as all basically nest in burrows but forage above ground, including some in desert environments during the day. I was also surprised to see volant species as "buffered" given that metabolic demands of flight are often cited as a reason bats are more sensitive to temperature. And how were aquatic/semi-aquatic species handled? It appears the semi-aquatic rodents were considered "buffered" but not semi-aquatic carnivores (Lontra canadensis and Neovision vison). For all of these, simply citing your reasoning/methods in a bit more detail may help explain them: for example, did you rely on specific citations? Identification of keywords in species accounts? One primary source then others to fill gaps? Genus-level descriptions? Was other information besides activity location used?. Without greater explanation as to how these categories were assigned, I have pretty strong concerns about this trait's results.

We took this comment by the reviewer seriously and have reassessed every life history trait assignment for each species. We have also reviewed substantial, additional literature for each species and trait, which are included in our table for future researchers to build upon. These exercises prompted changes to a few life history assignments, including for hibernation. In addition, we agree that our binary "buffered" category was too simplistic. We, thus, created a 3-state category which accounts for species that facultatively use habitat buffering, species that use obligate buffering, and species that do not use habitat buffering at all. We added in a "Supplemental Methods" section to the Supplemental materials, where we explain how we categorized species into different habitat buffering levels. We now provide life history traits, with sources, for each species in Supplemental Table 1, in addition to the updated information in Figure S2.

11. For hibernation ability (related to my comment from Line 128-129), the way you've done it seems adequate for a broad look at the trait, but I worry that by lumping together species that use daily torpor with annual obligate hibernators, you may be conflating two different strategies and therefore potentially different responses. At the very least, sensitivity analyses such as those for body size bins seem warranted, in which you further split these sub-groups of dormancy. Just among rodents for example, the body size response to temperature of some of the ground squirrels, jumping mice, and small heteromyids (e.g. genus Perognathus) that are completely inactive (or nearly so) for several months are likely affected by differences in their resource acquisition due to the length of the growing season during which they are active (See the Eastman et al paper, above). In contrast, species that go into daily torpor are doing so more strictly to evade challenging temperatures. (See Geiser, Fritz, and Thomas Ruf. "Hibernation versus daily torpor in mammals and birds: physiological variables and classification of torpor patterns." Physiological Zoology 68.6 (1995): 935-966.) Aestivation versus hibernation is another possible discrepancy in the broad trait of dormancy, though the relatively low number of mammals that aestivate makes it less likely to change your results I would guess. Note that this idea comes up in the Discussion as well (Line 365).

As with habitat buffering, we have further explained our hibernation categorization in the Supplemental Methods section and provided additional literature in support of our assignments. We agree with the reviewer that it is important to examine whether mammals that use daily torpor versus mammals that hibernate vary in body size in response to temperature. Similar to 'habitat buffering', and we therefore divided this category into three states, with species that hibernate, use torpor, or neither (see updated results, figures, and discussion).

12. Line 251 – The overall results may be largely concordant, but models not incorporating phylogeny ignore an important and known source of variance in the data (phylogenetic signal) and thus are invalid for drawing conclusions. It is therefore more appropriate to report, interpret, and discuss the phylogenetic corrected models in the main text and put the “traditional” or non-phylogenetic contrast methods in the Supplemental. Moreover, by accounting for the variance due to phylogeny, the PGLMM model results cut more directly to your main objective: isolating the ecological patterns (above and beyond the effect of phylogeny and other non-focal variables, such as sex, season, etc.). For this dataset especially, which span deeply divergent lineages, accounting for phylogenetic relationships seems critical. Furthermore, all of the traits in question have often been shown to have moderate to strong phylogenetic signal across mammals or within some of the orders or families studied, so phylogeny cannot simply be ignored.

This is a good point. We initially had the non-phylogenetic models in the main text for ease of reader interpretation. However, we agree with the reviewer that it is more appropriate to move the PGLMMs to the main text and the LMMs to the supplemental material. The main text, including tables and figures, have now been updated in the manuscript to present the results of the PGLMMs. We updated the discussion to reflect these changes as well. The LMM results are now in the supplemental material (Table S2).

RESULTS
13. Table 1. I find it very difficult to easily compare the results for mass vs HBL as currently formatted. Could A and B be aligned next to each other? Also, the β symbol should be included in the Table header to more clearly link the text to the table. Same comments for Table S3.

We updated the tables as such.

14. In all Figures, it should be clear that the temperature values are on a standardized scale, not raw temperatures. Unless the US is just much, much colder than I realize. :)

We have now provided this information in the figure legends.

DISCUSSION
15. Line 307-308 – I think your results present strong and compelling evidence for the effect of population density, but it’s not clear on what evidence you make the claim that population density is more tightly linked to body size than temperature. Don’t your results indicate that it depend on the metric of body size? If you are basing this on the fact that the main effect of MAT is only significant for 1 metric (mass), I would note that both are significant in your PGLMM model, which is the valid set of results to interpret. And the magnitude of the main effects is still opposite for HB versus mass. A version of this statement which appears in the abstract should also be revised unless there is other evidence presented to support the claim.

We agree with the reviewer’s summary of the results, especially when presenting the PGLMM model results in the main text. We have updated the text in the abstract and discussion to indicate that both urbanization and temperature were important predictors in our study.

16. Lines 371-374 – Excellent point. There are a couple good examples out there you may choose to cite: The Eastman et al 2012 paper looks at exactly that, as well as a brand new paper that may be nicely complementary to many of the ideas you present: E. A. Riddell, K. J. Iknayan, L. Hargrove, S. Tremor, J. L. Patton, R. Ramirez, B. O. Wolf, S. R. Beissinger. Exposure to climate change drives stability or collapse of desert mammal and bird communities. Science. 05 Feb 2021 : 633-636.

We thank the reviewer for the suggested citations, which are highly relevant. Both have been added to this section.

Reviewer #2 (Remarks to the Author):

In their manuscript, *Mammalian body size is determined by interactions between climate, urbanization and life history traitis,* Hantak et al. set out to test how climate, and urbanization interact with species traits to determine body size in a over 100 mammal species. They conclude that urbanization is associated with larger size, suggesting resource availability is a stronger determinant of size than temperature. This effect is mediated by life history and size itself, with larger species and those with thermal buffering life history strategies being more sensitive to temperature change. This is a well laid out manuscript, addressing an interesting set of questions that are clearly outlined and compellingly justified. I have several fairly major concerns, but if the authors are able to address them, I think this could be an exciting and important contribution. I also have some minor suggestions.

We thank the reviewer for the interest in our study, and we appreciate the feedback, which improved the overall quality of this manuscript.

My main concerns are:

1) There is limited consideration of the mechanism by which temperature-driven size reduction may be happening. This has led to limited consideration of what temperature variable matters. Is the mean annual temperature of the cell where a specimen was collected really the temperature that would determine body size? Shouldn't it be the temperature in the location and at the time that reproduction occurred? I think this deserves much greater consideration both conceptually and empirically (e.g. through sensitivity analyses).

We used the highest resolution available for each variable in our dataset. We are not able to obtain temperature data from the exact time of reproduction or from specific geolocations of collected specimens. We were also not able to include temperature data from the time of most intense growth in each individual, due to a lack of detailed age data for each individual. However, to address the concern of mean annual temperature in our modeling framework, we took the approach of another reviewers suggestion (outlined above). Here, we ran models with mean temperature and precipitation across 2 years (collection year + 1 year before) and 5 years (collection year + 4 years before), in addition to our initial models with the year of sample collection. All results were consistent across the models with different combinations of annual temperature. We added new text to indicate that we checked the stability of our modeling approach in this way. Lines 238-241.

2) Given urban heat island effects, it seems like temperature and human pop density should be highly correlated. Given the complexity of the model, if this is the case, it seems like it might be hard to interpret the directionality of the relationships recovered in the model.

We checked model variance inflation factors (VIFs) and found no highly correlated single predictor variables in our model. We added this explanation to lines 246-247.

3) The treatment of time and size in the model seem unnecessarily imprecise. Why use a random effect for decade when you could include year as a fixed effect? Why not use mean body size for a species as a predictor rather than binning the data?

In this study, we were interested in modeling spatial, not temporal, body size tends. We used the random decade term to control for broad-scale temporal variation within our models without making explicit

assumptions about yearly change. In addition, yearly and decadal trends across our predictors are not expected to vary from each other substantially, especially compared to trends across the entire length of the time series used in this study.
We binned body size data rather than use mean body size as a predictor because we obtained the average size of the mammals from our dataset.

4) I am of the opinion that this paper should not be published without explicit acknowledgement of each of the museums that house the specimens used; I recommend acknowledging the institutions at the least, and the curators-in-charge and collections managers from mammalogy if possible. Given the scope of the dataset, this could be done in supplemental materials.

We agree that we should directly acknowledge the institutions, and have done so now in the acknowledgements. We wish the data citation ecosystem was more robust and could support direct citations of data publishers for increased impact and usage tracking by museums themselves. If Communications Biology allows, we would be willing to have a supplemental citation section that cites each data provider. We have not done this yet, but have added institutions to acknowledgements and will see what the Editor says about the supplemental citation idea.

5) Assuming the effect sizes are comparable, the effect of MAT is higher than the effect of population density; this seems at odds with the framing of the paper, that urbanization is more tightly linked to size than temperature. Apologies if I've misinterpreted this somehow.

We agree with the reviewer, especially when presenting the PGLMM model results in the main text. We have updated the text in the abstract and discussion to indicate that both urbanization and temperature were important predictors in our study.

Minor concerns:
Line 145: Does total body length include tail? Is tail thermoregulatory, and could it be increasing as a result of Allen's Rule? Rather than reference Guralnick (2016) here without any detail; could you include enough so that people generally know what you did without being familiar with the reference? It seems like – given the later text – tail is excluded; this would be worth clarifying throughout.

Yes, total body length includes the tail, but we were specifically interested in head-body length, which is not a measure in VertNet and one which we calculated. To obtain measures of head-body length, we subtracted total length from tail length. The Guralnick et al (2016) reference is for the trait extraction process from VertNet, which also details and justifies our use of the head-body metric, and we believed would not be necessary to re-explain in our paper.

Line 149 – How did you determine there were no systematic biases?

We examined body mass variation across sources and found no obvious deviance. We added this explanation to lines 143-144.

Why was population density log transformed for the modeling?

We log transformed human population density to normalize data. We repeated this information in the "mixed-modeling framework" section with more detail. We thus removed the first instance that this was mentioned.

Why bin body size (Line 219) instead of using average size per species as a predictor? Why not include Year as a fixed effect rather than decade as a random effect?

See response to major comment #3.

Why present the LMM results rather than the PGLMM results? I imagine the dredging is not possible with PGLMM, so it makes sense to do it with the LMMs, but once you have the best model, it seems like it makes sense to account for phylogeny.

We agree with the reviewer and have updated the main document to present the PGLMM results and moved the LMM results to the supplemental material.

I think you should present the correlations among the predictors as a supplemental table.

We now present the predictor correlations for body mass and head-body length datasets as supplemental figures (Figs S3 & S4).

Typos in the Table 1 legend – (A) HB length

Fixed.

The Introduction is light on references – see for example, the beginning of the paragraph that starts on Line 86.

Based on suggestions from all reviewers, there have been a number of new relevant references added to the introduction.

Reviewer #3 (Remarks to the Author):

In this paper, Hantak and colleagues investigate variation in mammalian body size across the United States in relation to both climate variables and human population density (proxy for urbanization). The authors also investigate how traits such as diel activity patterns, hibernation ability, and ability to "buffer" their habitat (e.g., via burrowing) influence these patterns in body size. The authors compiled a very large dataset of mammals and explored these relationships using linear mixed models. They found that, surprisingly, urbanization has a larger effect on mammalian body size than temperature and that mammals tend to be larger in urban environments. The authors also find that different life history traits also modulate these patterns, and they discuss implications for the dual effects of climate change and ongoing urbanization on mammal populations in the future.

I enjoyed this paper and think that the methods the authors used were appropriate to explore the questions proposed. The results are novel and should be of broad interest to conservationists, climate change researchers, mammalogists, and ecologists. I only had a few minor critiques, but these are mostly related to reorganizing certain sections or justifying certain methodological decisions.

We greatly appreciate the feedback on our manuscript, and below we carefully address each comment. Importantly, we feel that the revisions have greatly improved the quality of this manuscript.

(1) The authors should consider reorganizing the Introduction. The Abstract and Discussion are very well organized and have a logical flow that I was able to follow and anticipate the forthcoming section. In contrast, the Introduction felt a little jumbled and I struggled to follow from one point to the next. I offer specific suggestions in the comments-by-line below. In general, clearly setting up the major variables explored in this study and what the rationale was for investigating them will greatly improve the paper.

We thank the reviewer for the suggestions on how to facilitate readership of the introduction. We explain the changes that were made to the manuscript in the specific comments below.

(2) Somewhat related to the Introduction, I found certain definitions/terminology to be used inconsistently in the paper or to not be clearly defined. For example, “life history traits”, “buffering traits”, “thermal tolerance traits”, or simply “traits” are often used interchangeably, or at least without sufficient definition of how they differ. I’ve left specific comments below, but in particular the idea of “buffering” and how one defines a “buffering” vs. “non-buffering” mammal would be helpful.

We agree with the reviewer that greater consistency was needed in terminology. We more carefully clarified the traits of interest throughout the manuscript. We also included more information in the methods section and in the supplemental materials.

(3) In the figures/tables a more thorough description of the data being presented would be very helpful, especially for future readers who may be skimming through. Related to that, the comparison between Table 1 and Supplemental Table S3 would be much improved if the same format/columns were used for both (or if that is impossible some explanation as to why).

We added more information to the table and figure legends and formatted the LMM and PGLMM results tables as similarly as possible.

Below, I list my specific comments/suggestions. In general, I think the paper is well-written, well-conceived, and interpreted appropriately. In particular, I found the Discussion to be very well-written and concise. All of my comments should be considered minor, and I look forward to seeing this paper published in Communications Biology in the near future.

We appreciate the positive feedback and thank the reviewer for their time.

Sincerely,

Lucas N. Weaver

Comments by Line

Introduction

45: “understanding drivers of body size” sounds a bit odd, consider changing to “understanding drivers of body size variation” or something comparable.

Done.

45–46: Consider adding some relevant citations to make your point that understanding the drivers of body size variation has been a central goal of ecology over the last half century.

Relevant citations have been added to support this sentence.

47: Most readers will no doubt be familiar with “Bergmann’s Rule,” but it would be worthwhile to briefly summarize his findings before adding the caveat statement, “…although with much debate…”

We added in a short explanation of Bergmann’s Rule.

54: “While” is a time word, e.g., “While I was at the store my friend prepared dinner.” Consider changing to “Whereas.”

Done.

69: The authors state that heat island effects have been observed in “various animal taxa” but the only reference provided is for insects. Consider adding additional references to make this point, or specify that the pattern is most readily observed in insects.

This trend has largely been found in insects. We made this clearer in the text and added another citation to support this statement. Lines 71-73.

70: “…but limited support endotherms” is an incomplete sentence fragment. Consider revising to something like, “…but there is little support for heat island effects on endotherms.”

We fixed this sentence by adding “in” before the word ‘endotherms’. Lines 71-73.

70–71: The sentence that begins with, “Little attention…” seems out of place given the preceding sentence, and I am not sure it is necessary. The next point the authors make regarding the previous study on deer mice follows well without this intervening statement. Consider removing or making a segue between the two points.

We agree this sentence was unnecessary and was, therefore, removed.

76: Here the authors use a comma after “i.e.” but elsewhere in the manuscript “e.g.” and “i.e.” are not followed by a comma. Consider keeping this consistent throughout.

Done.

76: The use of “Second” to begin the next portion of this paragraph implies there was a “first” but as constructed in is unclear what the “first” point was. I assume it is the observation that head-body length decreased but body mass did not in urbanized environments? The authors might consider reorganizing this paragraph to make their main points clearer. For example, a new paragraph could be started after the “limited support for endotherms” statement, then the authors could begin with a topic sentence outlining the main points they’ll make in the paragraph. Something like, “Previous studies have hypothesized that urbanized environments may influence mammalian body size by (1) selecting for individuals that are able to avoid detection (i.e., crypsis), (2) providing greater food and water resources (i.e., resource rule), and (3) favoring changes in life history traits. First…etc.” As currently written, it does not become clear that the authors are introducing hypotheses and predictions they aim to test in this study until the very end of the paragraph.

Yes, the first (albeit long-winded) point was that urban heat island effects may influence body size. We have three hypotheses of how body size may change across urbanization gradients. These are, urban heat island effects, resource availability, and fragmentation. We agree with the reviewer that these three points, were not clearly laid out as hypotheses. We amended this paragraph to make these three points clearer by reducing the text and rewriting portions of this paragraph. Lines 65-80.

86–89: This first sentence is a hard to follow. Consider revising in a similar way as suggested in my previous comment. I agree that mammals are a good system for investigating the questions of interest to this study, but the reasons why can be more clearly outline in a topic sentence, then expanded upon in the

meat of the paragraph, then re-emphasized at the end. For example, "Mammals represent a good study system for investigating how urbanization affects body size and life history traits because mammals are ecologically diverse, exhibit a wide range of body sizes, have diverse life history strategies, and are well-represented in biodiversity datasets." Or something like that.

We like this suggested change and have largely adapted it. Lines 81-84.

89–91: Consider adding some citations to support the statement that "Mammals have evolved to fill…etc." To most readers this is probably obvious, but a few references would be useful to guide folks who might be less familiar with mammalian diversity/ecology. Even if it is a broad reference text like Walker's Mammals of the World (Nowak, RM, 1999, Johns Hopkins University Press).

We incorporated the suggested citation, but opted not include more based on the generality of the statement. Lines 84-86.

91–92: Consider providing a reference(s) for the statement, "These traits are expected to strongly mediate…etc." If there are no previous studies that support this point, consider revising to say something like, "We expect that these traits strongly mediate….etc."

We revised this sentence based on the reviewers second suggestion. Lines 86-87.

96–97: I'm not sure the preceding statement explains why habitat buffering might lessen the effect of urbanization on mammalian body size. This also seems a bit out of place in a paragraph about why mammals make a good study system. Consider either moving these thoughts into the preceding paragraph, or making it clear why mammals have better "buffering" capabilities compared to other organisms and why this might dampen the effects of urbanization on body size.

We agree that this statement was out of place in the current paragraph. We have removed it, as we explain our life history trait predictions in the last introduction paragraph.

103–105: Consider mentioning from where this dataset was derived (e.g., compilation from primary literature) and also citing Supplemental Table S1 since it provides the references for each species. This is an impressive dataset!

We added in the reference to Supplemental Table S1, but we did not mention what the data sources were because this information would be redundant with the first sentence in the Methods sections, which explains the sources in detail.

105–107: Consider rewriting the sentence, "Our overarching question…etc." As currently written, it is a bit convoluted. For example, the explanation of what you mean by "human population density" could be put in the next sentence instead of placed in the middle of what is an important statement of your primary goal(s).

We agree with the reviewer. This sentence has now been simplified. Lines 98-100.

103–123: This is a great paragraph outlining the questions/hypotheses you'll be exploring in this paper. I think reorganizing the preceding paragraphs slightly as I suggested above will help guide the reader more naturally to these ideas.

We agree that the previous suggestions dramatically aided in the readership of the introduction.

124–128: Is there a reason the authors chose to refer to traits such as fossoriality, the ability to hibernate, and diel activity pattern as life history traits rather than ecological traits? Typically, I think of life history traits as being related to development and reproduction (e.g., growth rate, reproductive timing) rather than the way in which an animal interacts with its environment. This is not a major issue, and perhaps is a more common practice than I am aware of, but the authors might consider explaining/justifying their choice to refer to these as “life history traits”.

We agree with this point and changed the terminology to “ecological traits” – this helps keep “life history traits” better circumscribed and reduces confusion in the literature.

Methods

195–197: The authors might consider clarifying why human population density, compiled from a source with 1x1 km resolution chose to aggregate into 10x10 km bins. Further, they might consider explaining what they mean by “appended to each record.” I assume that the records are the mammalian occurrence records and that the reason for aggregating the more precise human population data into larger bins is due to the lower spatial resolution of the mammalian occurrence data, but this could be more clearly spelled out here.

Our rationale is two-fold. As the reviewer notes, our records are often not as spatially precise as 1km grain. As well, we are explicitly interested in context of human population density, and thus a more aggregated view is reasonable to examine that human population density context. Re: “appended to each record” just means we annotated specimen records with the human population values related to locations where they were collected. We have made small wording adjustments to improve clarity in the main manuscript.

198–199: Does the discrepancy in climate data resolution (4 km) and human population density resolution (10 km) pose any problems for downstream analyses?

No, this is not problematic. See reply above.

215–216: Were your life history traits determined from the same sources listed at the beginning of the Methods section? If so, consider mentioning that here, and if not consider adding a brief statement of where these data came from.

All of the life history references can be cross-referenced with Table S1 and the Supplemental References section. We have now made this more clear in the text. Lines 208-219.

217: For habitat buffering the authors present the binary “fossorial vs. terrestrial” as an example. The authors should consider listing all of the habitat buffering traits they used here, or at least point the reader to where they could find them (I may have missed something, but can’t find it listed anywhere). Also, does “terrestrial” refer to all non-burrowing mammals (e.g., arboreal)? The authors should consider clarifying what these bins refer to, keeping in mind that many mammals that are considered “terrestrial” can also burrow (e.g., rats). Issues like these are unavoidable in large datasets like those used in this paper, but some appropriate caveats should be given nonetheless.

We updated this section to provide more information on the mammal life history traits. In addition, we now provide detailed descriptions of life history traits that are harder to define in the Supplemental Methods section. We also provide the life history trait categories and refences for each mammal species in Supplemental Table1.

Results

265: The use of "Traits" to start this paragraph is referring to what the authors consider "life history traits," correct? If so, consider specifying; traits can mean anything in this context (e.g., body mass is a "trait").

Yes, life history traits (now "ecological traits"). This has now been updated in the manuscript.

267–269: Doesn't this sentence contradict the previous statement? Hibernation was one of the traits considered to be a habitat "buffer" correct? If there is a substantial difference between hibernators and other "buffer" types (e.g., burrowing) that should be noted? In general with this section, the authors should consider being precise and consistent in their terminology so as to avoid confusion. This issue persists in the next sentence about diurnal vs. nocturnal species, which I took to represent another form of "buffering".

Hibernation and habitat buffering were two different life history traits used in this study. We paid careful attention to clarifying this point within the manuscript We also made sure to standardize our terminology throughout the manuscript.

Discussion
295–305: This paragraph is a great summary of the rationale behind the paper. Following a structure such as this would be useful in the Introduction; the authors might consider using this as a guide for how to more clearly set up the study early on.

Thank you. With the suggested revisions form all reviewers, we believe the introduction is now better organized.

Figures/Tables

Fig. 1: Great figure summarizing the spatial coverage and abundance of your data. Consider defining the abbreviation HB at the beginning of the figure caption rather than at the end.

Done.

Fig. 2: Consider adding to this figure caption an explanation of how this plot was generated.

This standalone figure was removed from the manuscript. Code for generating figures for this paper will be uploaded to Github.

Table 1: Consider explaining in the caption where the data for different columns were derived from (such that someone skimming the paper could get a sense of your analyses).

The table has been updated and reformatted.

Supplemental Information

Fig. S1: In the Introduction the authors mention that differences in areas sampled could influence your results (e.g., depauperate sampling of the southeast) but this point is never brought up again, or perhaps I missed it. Since you have this figure you might consider referring to it and adding some discussion about sampling gaps, perhaps in the Methods.

We now refer to this figure in the Methods. Lines 199-200.

Fig. S2: In this caption is the first reference I've seen for how you've defined your "habitat buffering mammals" beyond the "fossorial vs. terrestrial" example. These should be more clearly defined and justified in the Methods. That fossorial and subterranean taxa can "buffer" their environments through burrowing makes sense to me, but I would not consider volant taxa (i.e., bats) to buffer their habitats through flying. I'm sure the authors have some rationale for making this assignment, but I am not familiar with it and I imagine the same would go for other readers.

We agree with the reviewer that this information was lacking in our initial submission. Based on another reviewer's suggestion, we created more levels for the hibernation and habitat buffering categories (updated throughout the manuscript). We also added in a "Supplemental Methods" section to the Supplemental Materials, where we explain how we categorized species into different hibernation and habitat buffering levels.

Table S3: Is there a reason why the data reported for the PGLMM results are in a different format from the table in the primary text? This table is missing the standard error and p-value columns present in the other table. It would be helpful if the reader could directly compare the two results to see what effect phylogeny has on the modeling results. The congruence between the two models is pretty striking, especially given the dominance of rodents in the sample (which is to be expected), so I think providing that direct comparison would make your case even stronger.

We now focus on the PGLMM results in the main text of the manuscript. We standardized the PGLMM and LMM results table formats as closely as possible.

REVIEWERS' COMMENTS:

Reviewer #1 (Remarks to the Author):

Summary Comments

The authors have addressed all of my previous concerns in the revised manuscript, “Mammalian body size is determined by interactions between climate, urbanization, and ecological traits”. Their responses to all reviewer concerns and the corresponding changes to the manuscript and supplemental materials are excellent. The improved explanation of species trait assignments and full reporting of those in the Supplementary not only eliminated the previous uncertainty in this paper’s methods but will also serve as an important resource for future trait-based mammal work. The authors diligently and thoughtfully made many helpful wording changes, added suggestion citations and acknowledgment of alternative explanations that have greatly improved the clarity and flow of the paper. I especially appreciate the authors’ ability to concisely contextualize and acknowledge the complexity of the factors studied so as not to over-interpret their results in the Discussion, while still clearly demonstrating many interesting and important relationships that I’m certain will be a source of inspiration for more studies for decades to come.

Minor comments

Line 62 – did Ives et al look at all taxa? Plants? All vertebrates? Birds (which are arguably much more mobile)? Mentioning the taxon of these threatened species here would be helpful because it is such a counterintuitive (but fascinating) point.

Figure 2 and 3. The 95% credible intervals are no longer present and should be re-added to each plot. Also, this is personal preference and primarily for ease of interpretation, but I recommend ordering each plot legend in the most intuitive order. For example, Human Pop. Density would be best as Low, Mid, High rather than high, low, mid as it is now. Same for the states of hibernation, etc, which are sort of ordinal.

Reviewer #2 (Remarks to the Author):

The authors' revisions have thoroughly addressed all of my concerns - I see no other issues, and thank them for taking the time to consider my comments. Congratulations on this exciting work, and I look forward to seeing this in print.

Reviewer #3 (Remarks to the Author):

I reviewed an earlier draft of this manuscript and had only minor suggestions for improvement, mostly related to the organization of the Introduction and the definition/justification of variables such as 'habitat buffering.' The authors have satisfied all of my minor issues. I believe this paper is very well written, well illustrated, and appropriately interpreted. I have no further suggestions or issues and recommend that it should be published in Communications Biology. Congratulations to the authors on this impressive work.

REVIEWERS' COMMENTS:

Reviewer #1 (Remarks to the Author):

Summary Comments

The authors have addressed all of my previous concerns in the revised manuscript, “Mammalian body size is determined by interactions between climate, urbanization, and ecological traits”. Their responses to all reviewer concerns and the corresponding changes to the manuscript and supplemental materials are excellent. The improved explanation of species trait assignments and full reporting of those in the Supplementary not only eliminated the previous uncertainty in this paper’s methods but will also serve as an important resource for future trait-based mammal work. The authors diligently and thoughtfully made many helpful wording changes, added suggestion citations and acknowledgment of alternative explanations that have greatly improved the clarity and flow of the paper. I especially appreciate the authors’ ability to concisely contextualize and acknowledge the complexity of the factors studied so as not to over-interpret their results in the Discussion, while still clearly demonstrating many interesting and important relationships that I’m certain will be a source of inspiration for more studies for decades to come.

We appreciate the positive feedback from the reviewer and thank them for their constructive suggestions and edits.

Minor comments

Line 62 – did Ives et al look at all taxa? Plants? All vertebrates? Birds (which are arguably much more mobile)? Mentioning the taxon of these threatened species here would be helpful because it is such a counterintuitive (but fascinating) point.

All taxa. We more explicitly state this by changing “species” to “plants and animals”.

Figure 2 and 3. The 95% credible intervals are no longer present and should be re-added to each plot. Also, this is personal preference and primarily for ease of interpretation, but I recommend ordering each plot legend in the most intuitive order. For example, Human Pop. Density would be best as Low, Mid, High rather than high, low, mid as it is now. Same for the states of hibernation, etc, which are sort of ordinal.

We added 95% credible intervals to figures 2 and 3. And as the reviewer suggest, we changed the order of the plot legend for human population density.

Reviewer #2 (Remarks to the Author):

The authors' revisions have thoroughly addressed all of my concerns - I see no other issues, and thank them for taking the time to consider my comments. Congratulations on this exciting work, and I look forward to seeing this in print.

We thank the reviewer for their positive feedback and helpful suggestions.

Reviewer #3 (Remarks to the Author):

I reviewed an earlier draft of this manuscript and had only minor suggestions for improvement, mostly related to the organization of the Introduction and the definition/justification of variables such as 'habitat

buffering.' The authors have satisfied all of my minor issues. I believe this paper is very well written, well illustrated, and appropriately interpreted. I have no further suggestions or issues and recommend that it should be published in Communications Biology. Congratulations to the authors on this impressive work.

We thank the reviewer for their time and constructive suggestions.
